# The effects of microglia on tauopathy progression can be quantified using Nexopathy *in silico* (Nex*is*) models

**DOI:** 10.1038/s41598-022-25131-3

**Published:** 2022-12-07

**Authors:** Chaitali Anand, Pedro D. Maia, Justin Torok, Christopher Mezias, Ashish Raj

**Affiliations:** 1 Department of Radiology, University of California San Francisco, San Francisco, CA USA; 2 Department of Mathematics, University of Texas at Arlington, Arlington, TX USA; 3 Department of Neuroscience, Cold Spring Harbor Laboratory, Cold Spring Harbor, NY USA

**Keywords:** Alzheimer's disease, Computational models, Computational neuroscience, Microglia

## Abstract

The prion-like transsynaptic propagation of misfolded tau along the brain’s connectome has previously been modeled using connectome-based network diffusion models. In addition to the connectome, interactions between the general neurological “milieu” in the neurodegenerative brain and proteinopathic species can also contribute to pathology propagation. Such a molecular nexopathy framework posits that the distinct characteristics of neurodegenerative disorders stem from interactions between the network and surrounding molecular players. However, the effects of these modulators remain unquantified. Here, we present Nexopathy *in silico* (“Nex*is*”), a quantitative model of tau progression augmenting earlier models by including parameters of pathology propagation defined by the molecular modulators of connectome-based spread. Our Nex*is*:microglia model provides the first quantitative characterization of this effect on the whole brain by expanding previous models of neuropathology progression by incorporating microglial influence. We show that *Trem2*, but not microglial homeostasis genes, significantly improved the model’s predictive power. *Trem2* appears to reduce tau accumulation rate while increasing its interregional spread from the hippocampal seed area, causing higher tau burden in the striatum, pallidum, and contralateral hippocampus. Nex*is* provides an improved understanding and quantification of microglial contribution to tau propagation and can be flexibly modified to include other modulators of progressive neurodegeneration.

## Introduction

Alzheimer’s disease (AD) is a progressive neurodegenerative disorder that exhibits extracellular amyloid-$\beta$ plaques and intracellular aggregates of hyperphosphorylated tau as its main neuropathological hallmarks. The regional location and density of tau tangles correlate more with the degree of clinical symptoms than those of amyloid-$\beta$ plaques^1,2^. The stronger association between tau and regional atrophy, hypometabolism, and cognitive decline in AD^3,4^ makes it imperative to shift the long-standing focus from amyloid-$\beta$ to tau and to further investigate modulators of tau accumulation and spread.

Although tau mainly spreads via the brain’s anatomic connectome, the complete characterization of tauopathies should be based on the notion of Molecular Nexopathy^5^, which states that *divergent spatiotemporal patterns of different tauopathies may result from biochemical differences between tau species and the neural cells in the brain that interact with tau as well as the connectome-based mechanism of transsynaptic transmission*^5^. To unpack this, even though the key mediatory role in tau spread is played by the connectome, there are many other interactions between cells, circuits, proteopathic species, and the general neurological milieu in the neurodegenerative brain that remains outside of the connectome-centric view of these diseases. Evidence for this notion has become stronger over years suggesting the existence of important condition-altering differences in pathological tau species between degenerative conditions. For instance, tau displays differential 3R and 4R isoforms^6^ and hyperphosphorylation sites^7^ in different tauopathies. Gross tangle structure also differs across diseases, with AD tau forming neurofibrillary tangles and pre-helical formations, Pick bodies in Pick’s disease, and progressive supranuclear palsy, corticobasal degeneration, and frontotemporal dementia presenting more abundant tangles^8^. Indeed, pathological tau transits with a different mechanism in a disorder-dependent manner^5,9^.

Tau propagates in a stereotypical pattern in AD, starting from the entorhinal cortex to the hippocampus followed by the neocortex as described by Braak staging^10^. Experiments involving the injection of synthetic tau fibrils in the mouse brain have demonstrated that tau can propagate from the injection site to distant but connected regions^11^. Tauopathy progression is prion-like - templated and self-propagating^12^, with pre-existing aggregates acting as seeds to induce further misfolding. Since tau primarily migrates along the white matter tracts of the brain, it is reasonable to model its spatiotemporal dynamics in a first-order approximation as a diffusive process between connected brain regions. Prior work by our group has quantified pathology progression in humans and animals using such an approach via a graph-theory based system of differential equations called the Network Diffusion Model (NDM)^13–15^. However, although the NDM framework considers the connectome graph as the central (sole) mediator of pathology spread along a networked representation of the brain, it fails to consider the impact of molecular nexopathic species on tau spread.

**Figure 1:**
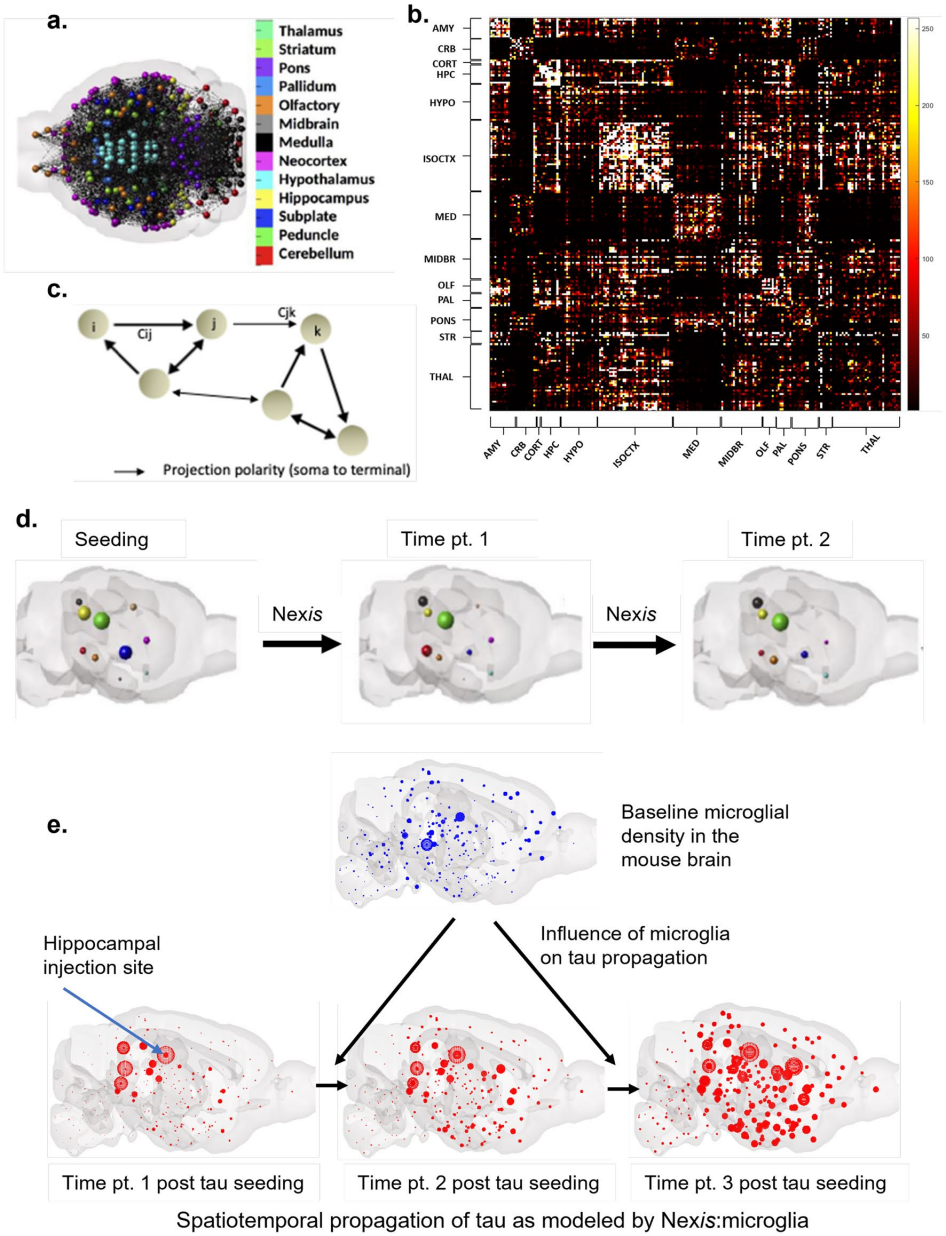
Modeling of tau propagation and the hypothesized influence of microglia. Panel (**a**): Representation of the brain as a network, with each ball indicating the center of mass of a region and the line between any pair of regions indicating a connection between them. Panel (**b**): Connectivity matrix of unilateral 213 cortical regions of the adult mouse brain. Panel (**c**): Graphic of the whole brain connectivity network with nodes (i,j,k) representing brain regions and connection strengths (Cij, Cjk) representing mesoscale connectivity. Panel (**d**): Illustration of Nex*is*-modeled progression of tau across time with each ball representing a region and ball-size indicating the degree of pathology burden. Panel (**e**): Per-region baseline microglial density is depicted in blue on the graphic of the sagittal mouse brain. Tau pathology at different time points post tau seeding is depicted in red on the mouse brains. The blue arrow on the glass brain at time point 1 indicates the regional injection site (hippocampus in this case). Panels (**a**), (**c**) and (**d**) are adapted from Mezias et al.^15^. AMY, amygdala; CRB, cerebellar region; CORT, cortical subplate; HPC, hippocampal formation; HYPO, hypothalamus; ISOCTX, isocortex; MED, medulla; MIDBR, midbrain; OLF, olfactory areas; PAL, pallidum; PONS, pons; STR, striatum; THAL, thalamic areas.

Here, we attempt to capture this nexopathy paradigm using mathematical *in silico* modeling, which we refer to as Nexopathy *in silico* or “Nex*is”*. Nex*is* models the following biological processes: trans-neuronal transmission along the connectome (modeled using the NDM); prion-like amplification of tau over time; and the nexopathic effects of extra-connectomic molecular players that modulate these processes. We formulate from first principles the process by which neuronal and other cells, genes, and other modulators might interact with misfolded tau during its accumulation and transmission along neural connections. Although these principles are qualitatively well-established from preclinical *in vivo* and *in vitro* studies, the unique benefit of formulating them in a mathematical model is that the model then becomes a means for testing interesting hypotheses and becomes a *testbed* for novel hypothesis generation. The overall design of Nex*is* and its typical workflow is depicted in Fig. 1a–d.

Important nexopathic effects are wielded by microglia^16^, the brain’s resident immune cells, and tau dynamics are well known to be influenced by the action of microglia often found in the vicinity of pathological aggregates. Recent studies of postmortem AD brains have revealed that microglia co-localize with tau and activate themselves after internalizing tau aggregates^17^. Tau-containing neurons can also activate adjacent microglia causing them to form junctions with other neurons as conduits to transfer tau and propagate pathology^18^. Using mouse models of rapid tau propagation, Asai et al.^19^ and others have demonstrated that microglia markedly contribute to the clearance, recruitment, and spread of pathology. Microglial activation and neuroinflammation accompany tau tangle formation and importantly, parallel AD clinical symptoms^20^. Specifically, activated microglia express the triggering receptor expressed on myeloid cells 2 (TREM2). *Trem2* is a well-known AD risk-gene^21^, with loss-of-function mutations (such as R47H and R62H) increasing the susceptibility to late-onset AD^22,23^ and being related to the rate of cognitive decline^24^. In vivo, *Trem2* over-expression in pathological conditions is related to the recruitment of microglia to pathological aggregates. Although, studies assessing the relationship between *Trem2* deletion and tau pathology and neurodegeneration have yielded variable results^21^, empirical clinical and preclinical data suggest that the modulation of tau propagation by the action of microglia^19,25,26^ is germane to the study of progressive tauopathies.

Owing to the microglial contributions, quantitative estimates based solely on connectivity and regional pathology levels, e.g. those from our prior NDM^13^, cannot recapitulate all aspects of pathology spread patterns in AD. Therefore here, we apply our novel Nex*is* framework to interrogate the modulatory effect of microglia on tauopathy progression, by introducing microglial signatures within the Nex*is* model (Fig. 1e). We explore microglial influence under two potential modes, whereby microglia alter: (1) the rate of intra-regional tau accumulation and (2) the rate of inter-regional tau spread. These effects are not mutually exclusive but have different biological and mathematical implications. We first apply the non-microglia-influenced Nex*is* model of tau aggregation and spread (i.e. Nex*is*:global) characterized solely by tau accumulation/clearance rate in addition to its diffusion along the connectome. This model is further extended into a microglia-based Nex*is*:microglia model to incorporate their effect on tauopathy progression. We apply these Nex*is* frameworks to data from mouse models of tauopathy^27^ using a 426-region mouse connectome^28^. By utilizing microglial homeostasis genes^29^ (first principal component of *P2ry12*, *Cx3cr1*, *Fcrls*, *Olfml3*, *Hexb*, *Siglech*, *Sox5*, and *Jun*) and an activated microglial signature (*Trem2*) gene^30^, we show that the inclusion of microglia in the model quantitatively improves its predictive power, with *Trem2* inclusion largely outperforming that of microglial homeostasis genes. We developed an optimization algorithm to infer model parameters. Results confirm and quantify reduction of tau burden (accumulation) by *Trem2* and increase in tau transmissibility to regions ipsi- and contralateral to the hippocampal seed area. Given the strain-specific nature of tau pathology, we verified that the Nex*is*:*Trem2* model parameters for datasets DS6 and DS9 also fit the pathology from the corresponding 1:10 dilution studies from Kaufman et al.^27^. We then explored *Apoe*, another putative modulator of tau propagation, and found that it does not contribute significantly to tau progression in the current datasets used. Nex*is*:microglia was also applied to some other microglial genes (*Sorl1*, *Cd33*, *Clu*, *Sirpa*, and *Aif1*) regulating phagocytosis and neuroinflammation in AD progression^31,32^.

Overall, the Nex*is*:microglia framework provides a more complete model of tau propagation. This study brings together empirical mouse tauopathy models^27^, the mouse mesoscale connectome^28^, and gene expression atlas^30^ to achieve the first quantitative study of microglial involvement in tauopathy propagation developed and validated at the whole-brain level.

## Results

### Association between spatiotemporal tau pathology and microglial spatial distribution

**Figure 2:**
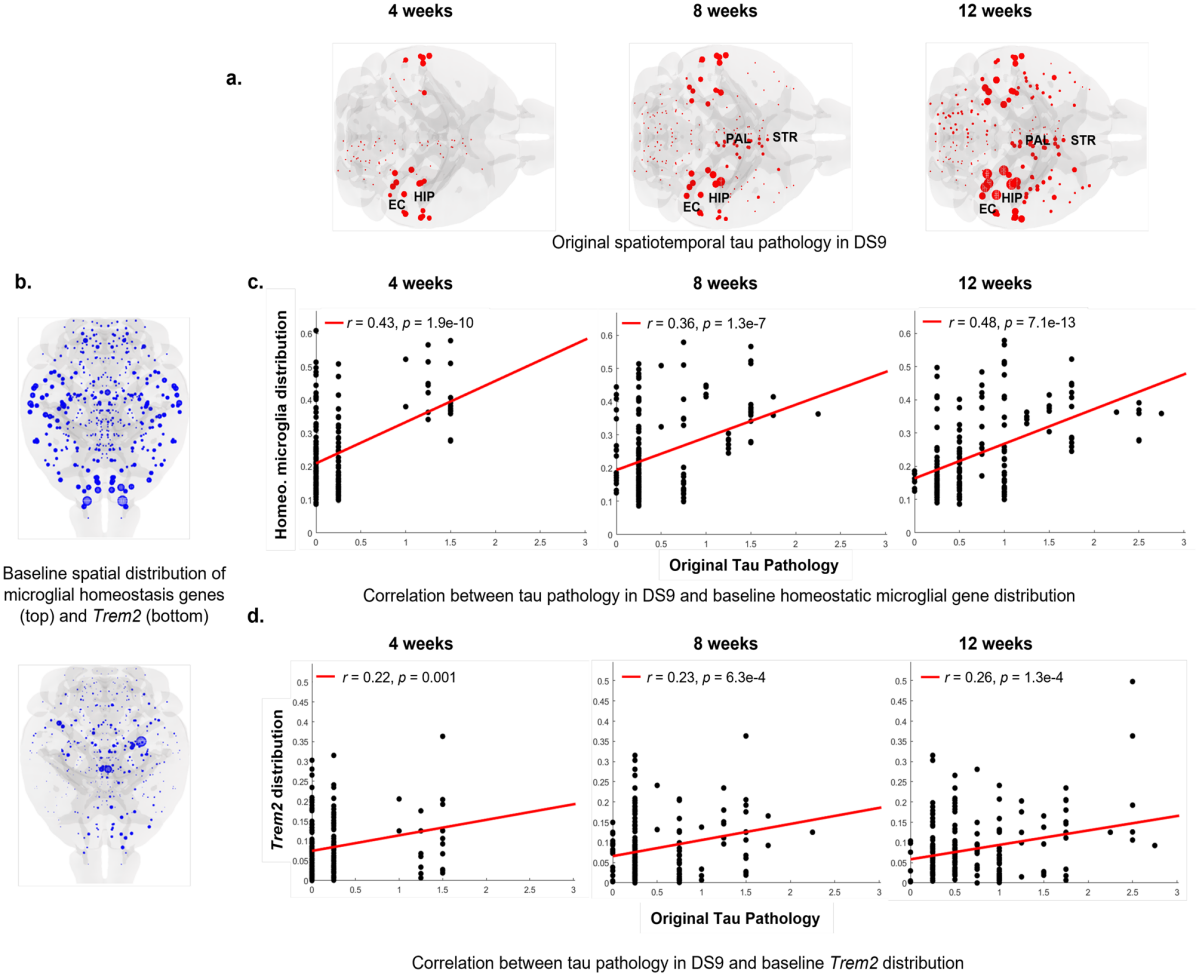
Correlation between spatiotemporal empirical tau pathology at 4, 8, and 12 weeks post hippocampal tau injection and baseline microglial gene spatial distribution for DS9. Panel (**a**): Empirical tau pathology in DS9 dataset post hippocampal seeding of exogenous tau^27^. Panel (**b**): Baseline spatial distribution of first principal component of microglial homeostasis genes and *Trem2* from a healthy adult mouse^30^. Panels (**c**) and (**d**): Correlation between spatiotemporal tau pathology progression at 4, 8, and 12 weeks post hippocampal seeding, and baseline expression levels of microglial homeostasis genes as well as *Trem2*. Sphere sizes in panel a correspond to the degree of tau burden in specific brain regions. HIP, hippocampus; EC, entorhinal cortex; PAL, pallidum; STR, striatum.

Correlations of tau pathology spread in sample dataset DS9 (Fig. 2a) with microglial homeostasis genes (Fig. 2b, c) as well as with *Trem2* (Fig. 2b, d) were significant, although modest. These data form a solid basis on which to undertake a more detailed and model-based exploration of the effects of microglia on tauopathy progression.

### Workflow of model implementation and validation

To implement and validate the Nex*is* models we chose whole-brain analysis approach with both the structural connectome and regional tau defined at the same parcellation scheme consisting of 426 regions (213 each hemisphere), each of which serves as a node of the connectome graph. Fig. 1a–c schematizes the network representation of the mouse brain for which we extract the connectivity matrix *C*. This connectome is the central (sole) mediator of tau spread in NDM (Fig. 1d). For Nex*is*, the analytical framework is augmented to account for the influence of tau accumulation/clearance rate (Nex*is*:global) followed by the influence of microglia (Nex*is*:microglia) (Fig. 1e). Since homeostasis genes and *Trem2* have distinct functional roles and display distinct baseline distributions (Fig. 2b), we chose to model them separately with two models following the Nex*is*:microglia framework: Nex*is*:homeostasis and Nex*is*:*Trem2*.

We validated the models at the whole-brain level on mouse tauopathy datasets DS4, DS6, and DS9 from Kaufman et al.^27^. Instead of pre-selecting certain brain regions and their tau levels as is usually done in such studies, we used all regional tau data, wherever available, in a completely unbiased fashion.

### Model parameterization depends on tau strain

The Nex*is*:microglia models include the following parameters: global accumulation/clearance rate of pathology, $\alpha$; global diffusivity rate constant denoting the diffusion of pathology between regions, $\beta$; accumulation/clearance of pathology as modulated by microglia, *p*; and pathology diffusion rate as modulated by microglia, *b*. Nex*is*:global is the non-microglial model with $p = b = 0$. The tau strains of the selected datasets were derived from mutually distinct sources: DS4 mice were injected with human AD-derived tau isolates, whereas DS6 and DS9 mice had tau derived from a P301S mouse and recombinant tau fibrils, respectively^27^. These pre-characterized datasets were made available by the Kaufman et al. study^27^ where they demonstrated distinct tau bio-activity marked by varying capacities to form and spread tau aggregates (seeding and spreading potential), widespread induction of pathology, and capacity to induce microgliosis. Figure S1 illustrates the distinct spatiotemporal patterns of tau pathology in DS4, DS6, and DS9 captured *in silico*.

**Figure 3:**
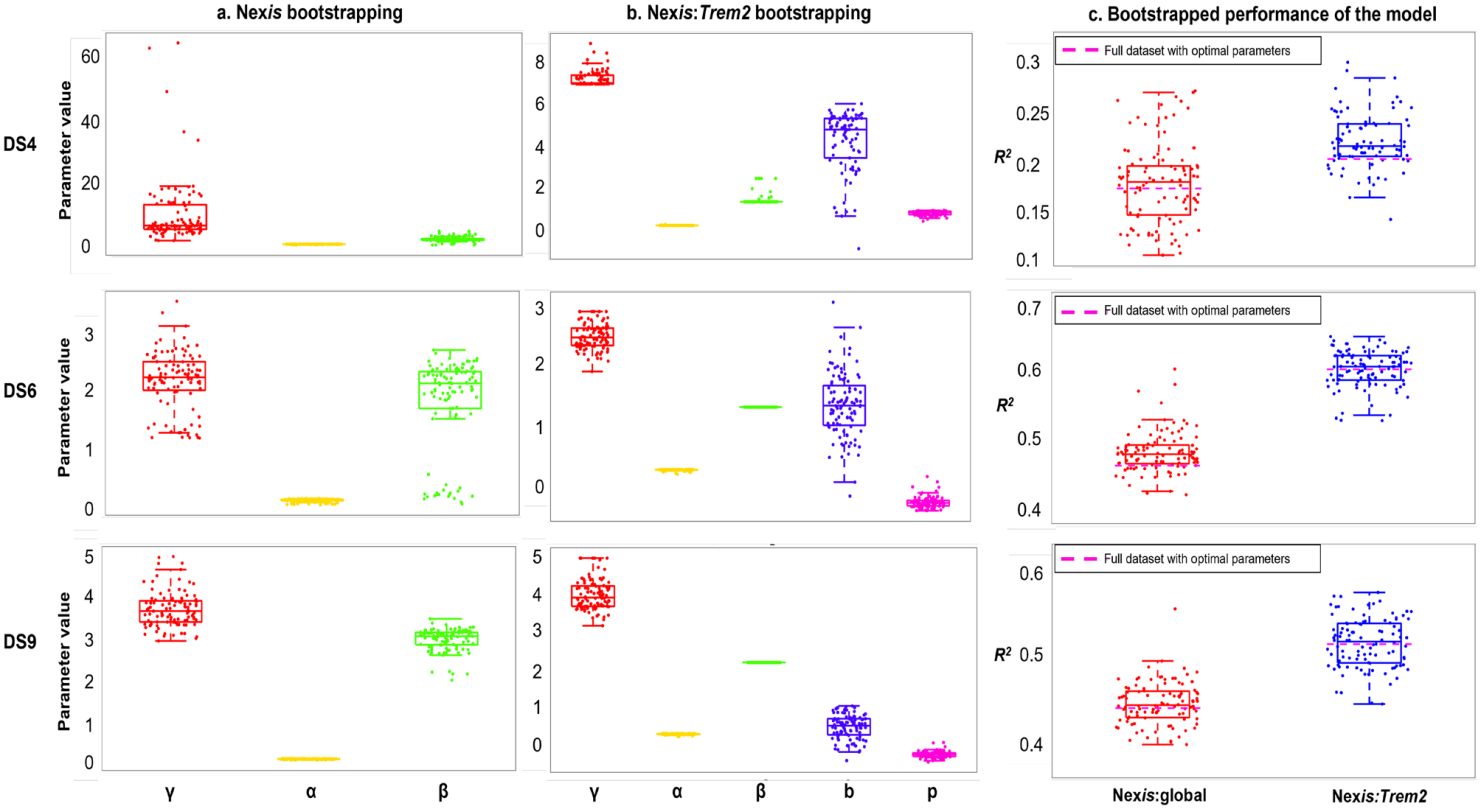
Mean and 95% confidence intervals for the global and microglial parameters as well as model fit for datasets DS4, DS6, and DS9. Panels (**a**) and (**b**): 100-iteration 80% resampled bootstrapped values of the Nex*is*:global and Nex*is*:*Trem2* model parameters. Panel (**c**): Comparison between model fits of the 80% resampled bootstrapped dataset with the full dataset (magenta dotted line). Global parameters: $\gamma$ (seed rescale), $\alpha$ (intra-regional tau accumulation rate), and $\beta$ (inter-regional tau spread rate). Microglial parameters: *p* (microglia-mediated intra-regional tau accumulation rate) and *b* (microglia-mediated inter-regional tau spread rate). Model fit is depicted by $R^2$.

**Table 1:** Comparison of model fits for NDM, Nex*is*:global, and Nex *is*:microglia (homeostasis and *Trem2*) for datasets DS4, DS6, and DS9.

Dataset	Model	Metric			Parameter value			
		$R^2$	AIC	BIC	$\alpha$	$\beta$	*p*	*b*
DS4	NDM	0.06	305.13	318.23	–	1.04 [0.00 to 4.26]	–	–
	Nex*is*:global	0.17	233.48	250.94	0.25 [0.05 to 0.30]	1.89 [0.05 to 3.84]	–	–
	Nex*is*:homeo	0.19	226.53	252.73	0.17 [0.17 to 0.18]	2.46 [2.46 to 2.46]	0.24 [0.16 to 0.29]	-0.62 [-0.87 - -0.42]
	Nex*is*:*Trem2*	0.20	216.41	242.61	0.17 [0.17 to 0.19]	1.42 [1.32 to 2.46]	0.77 [0.50 to 0.89]	4.25 [0.72- 5.79]
DS6	NDM	0.33	824.00	837.19	–	0.49 [0.03 to 1.90]	–	–
	Nex*is*:global	0.46	695.94	713.40	0.20 [0.13 to 0.23]	1.86 [0.23 to 2.61]	–	–
	Nex*is*:homeo	0.48	683.97	710.17	0.26 [0.26 to 0.26]	2.10 [1.30 to 2.41]	− 0.25 [− 0.34 to − 0.14]	− 1.05 [− 1.81 to − 0.13]
	Nex*is*:*Trem2*	0.60^***^	523.94	550.14	0.25 [0.21 to 0.26]	1.30 [1.30 to 1.30]	− 0.29 [− 0.42 to − 0.03]	1.33 [0.46 to 2.47]
DS9	NDM	0.26	701.20	714.30	–	0.68 [0.16 to 2.33]	–	–
	Nex*is*:global	0.44	545.09	562.55	0.19 [0.16 to 0.20]	3.01 [2.19 to 3.35]	–	–
	Nex*is*:homeo	0.45	533.88	560.13	0.24 [0.24 to 0.24]	3.91 [3.91 to 3.91]	− 0.22 [− 0.28 to − 0.18]	− 0.85 [− 1.25 to − 0.68]
	Nex*is*:*Trem2*	0.52^**^	463.64	489.84	0.22 [0.18 to 0.24]	2.10 [2.10 to 2.10]	− 0.31 [− 0.45 to − 0.10]	0.41 [− 0.21 to 0.92]

All Nex*is* model fits were significantly better than those of the NDM (*p* < 0.005). Nex*is*:microglia model fits are compared to the Nex*is*:global model fit (significance indicated by *). Significance of model fit comparisons is confirmed using the Fisher’s R-to-Z test. NDM: Network Diffusion Model, Nex*is*: Nexopathy *in silico*, AIC: Akaike’s Information Criteria, BIC: Bayesian Information Criteria, $R^2$: model fit parameter, $\alpha$: intra-regional tau accumulation rate; $\beta$: inter-regional tau spread rate; *p*: microglia-mediated intra-regional tau accumulation rate; *b*: microglia-mediated inter-regional tau spread rate. *** *p* = 0.001, ** *p* < 0.005.

We implemented a robust strategy for parameter estimation using a 100 iteration bootstrapping approach on 80% resampled data, allowing us to acquire 95% confidence intervals around the parameter means and to assess reproducibility. Table 1 summarizes the inferred parameters and quality-of-fit metrics. The results of parameter estimation using the bootstrapping approach (Fig. 3) include the mean and 95% confidence intervals for the model fit, global, and microglial parameters for Nex*is*:global and Nex*is*:*Trem2* of DS4, DS6, and DS9. Non-zero *p* and *b* values demonstrate microglial influence on tau accumulation and spread above and beyond Nex*is*:global. When *Trem2* microglial effects were included, DS6 and DS9 indicated enhanced clearance (*p* < 0) but increased inter-regional spread of tau (*b* > 0). Homeostatic microglial effects, however, led to lower inter-regional spread in DS6 and DS9 (*b* < 0 for DS6 and DS9; Table 1). DS4 demonstrated increased tau accumulation and spread due to *Trem2* compared to homeostatic microglia (Table 1).

### Inclusion of microglia improves the predictive power of Nex*is*

The NDM performed weakly compared to Nex*is* models for all datasets (Table 1). Nex*is*:global performed weakly for DS4 ($R^2$ = 0.17) but well for DS6 and DS9 ($R^2$ = 0.46 and 0.44, respectively), successfully recapitulating the spatiotemporal profile of empirical tau pathology. This suggests that passive network diffusion is insufficient and additional aggregation processes enhance the model’s predictive power.

The inclusion of microglial gene expression demonstrated higher predictive power ($R^2$) compared to Nex*is*:global. The improvement was statistically significant (per Fisher’s-R-to-Z transform *p* value) for DS6 and DS9. *Trem2* imparted significantly higher predictive power compared to microglial homeostasis genes. It is important to note that across all datasets, AIC and BIC decreased upon the inclusion of microglia, indicating that the better fits achieved by Nex*is*:microglia were not simply due to the introduction of additional model parameters. Instead, these results reflect reduction in uncertainty in the global parameters, $\alpha$ and $\beta$, as indicated by their narrower 95% confidence intervals. The eight chosen microglial homeostasis genes are used in the model as their first principal component. The accumulation and spread parameters for these Nex*is*:microglia models were either negative, non-significant, or much lower than those obtained for Nex*is*:*Trem2* (Table S1).

**Table 2:** Effect of dilute lysates. Nex*is*:*Trem2* model parameters for $\alpha$, $\beta$, *p*, and *b* obtained for DS6 and DS9 were applied to Nex*is*:*Trem2* for 1:10 diluted versions of those dataset (i.e. DS6_110 and DS9_110).

Dataset	Model	Metric			Parameter value				
		$R^2$	AIC	BIC	$\gamma$	$\alpha$	$\beta$	*p*	*b*
DS6	Nex*is*:*Trem2*	0.60	523.94	550.14	2.48	0.25	1.30	− 0.29	1.33
DS6_110	Nex*is*:*Trem2*	0.33	228.11	254.31	1.49	0.25 (fixed)	1.30 (fixed)	− 0.29 (fixed)	1.33 (fixed)
DS9	Nex*is*:*Trem2*	0.52	463.64	489.84	3.89	0.22	2.10	− 0.31	0.41
DS9_110	Nex*is*:*Trem2*	0.29	2.42	28.62	2.08	0.22 (fixed)	2.10 (fixed)	− 0.31 (fixed)	0.41 (fixed)

Seed rescale parameter ($\gamma$) was allowed to be fit for DS6_110 and DS9_110. Nex*is*: Nexopathy *in silico*, AIC: Akaike’s Information Criteria, BIC: Bayesian Information Criteria, $R^2$: model fit parameter, $\gamma$: seed rescale parameter; $\alpha$: intra-regional tau accumulation rate; $\beta$: inter-regional tau spread rate; *p*: microglia-mediated intra-regional tau accumulation rate; *b*: microglia-mediated inter-regional tau spread rate. The word ’fixed’ in parentheses means that this parameter has been fixed to that obtained for DS6 and DS9 while running the model to fit only the $\gamma$ parameter in DS6_110 and DS9_110, respectively.

Further, we consider the datasets DS6_110 and DS9_110, which use the same injected tau strains as DS6 and DS9, respectively, but at 1:10 dilution^27^. We expected that the tau-strain-specific parameters $\alpha$, $\beta$, *b*, and *p* should be consistent between these experiments, but the seed rescale parameter, $\gamma$, should be lower. We found that by applying Nex*is*:*Trem2* on DS6_110 and DS9_110 after fixing the former parameters to their respective values for DS6 and DS9 and fitting only for $\gamma$, the model provides strong fits to the data with lower $\gamma$ values, as anticipated (Table 2).

### Effect of *Trem2* on tau burden and spread

**Figure 4:**
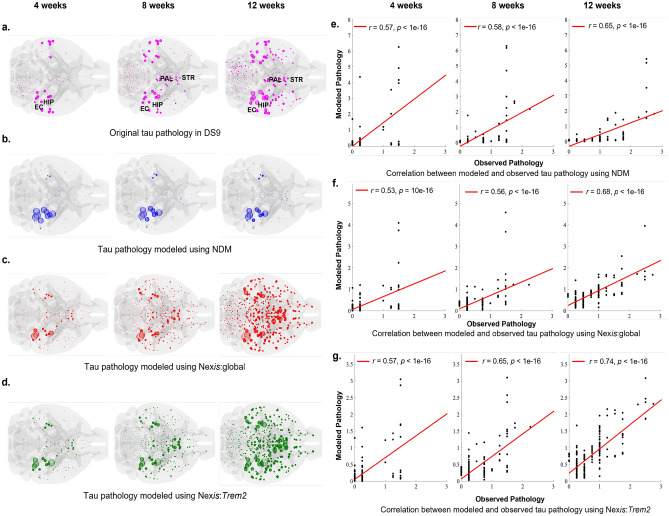
NDM, Nex*is*:global, and Nex*is*:*Trem2* model fits for dataset DS9. Panel (**a**): Empirical tau pathology observed in DS9 post hippocampal seeding of exogenous tau^27^. Panels (**b**)–(**d**): NDM, Nex*is*:global, and Nex*is*:*Trem2* modeled tau pathology progression, respectively, in DS9 dataset at 4, 8, and 12 weeks post hippocampal injection. Panels (**e**)−(**g**): Scatter-plots depicting the correlation between empirical and ND-modeled (**e**), Nex*is*:global-modeled (**f**), as well as Nex*is*:*Trem2*-modeled (**g**) tau pathology, respectively, at 4, 8, and 12 weeks post hippocampal tau seeding. Pearson *r* values are provided for each scatter plot. HIP, hippocampal area; EC, entorhinal cortex; PAL, pallidum; STR, striatum.

Since the fitting results highlight *Trem2* as the more significant modulator compared to homeostatic genes, the remainder of our results will focus on it. Figure 4 shows the results for DS9 in detail; they are very similar for DS6 (Fig. S3). For dataset DS4 (Fig. S2), while the Nex*is* models were significantly better than the passive NDM, improvement from Nex*is*:global to Nex*is*:microglia was not statistically significant (Table 1). Both Nex*is*-modeled distributions differ significantly from the NDM-modeled distribution. While both Nex*is*-modeled distributions look similar visually, there are clear differences between them that begin to become apparent at later time-points, especially in striatal and entorhinal areas. While both produce excellent recapitulation of empirical tau burden at all time points, the Nex*is*:*Trem2* model produces by far the better fits (Fig. 4f, g). Model fits in all cases improve over time as may be expected from a ramifying pathological process. The Nex*is*:*Trem2* fits incurred an increase in the microglia-dependent spread parameter, *b* (Table 1); hence *Trem2* appears to allow more pathology to efflux from the hippocampal seed regions at early time-points (Fig. 4d).

**Figure 5:**
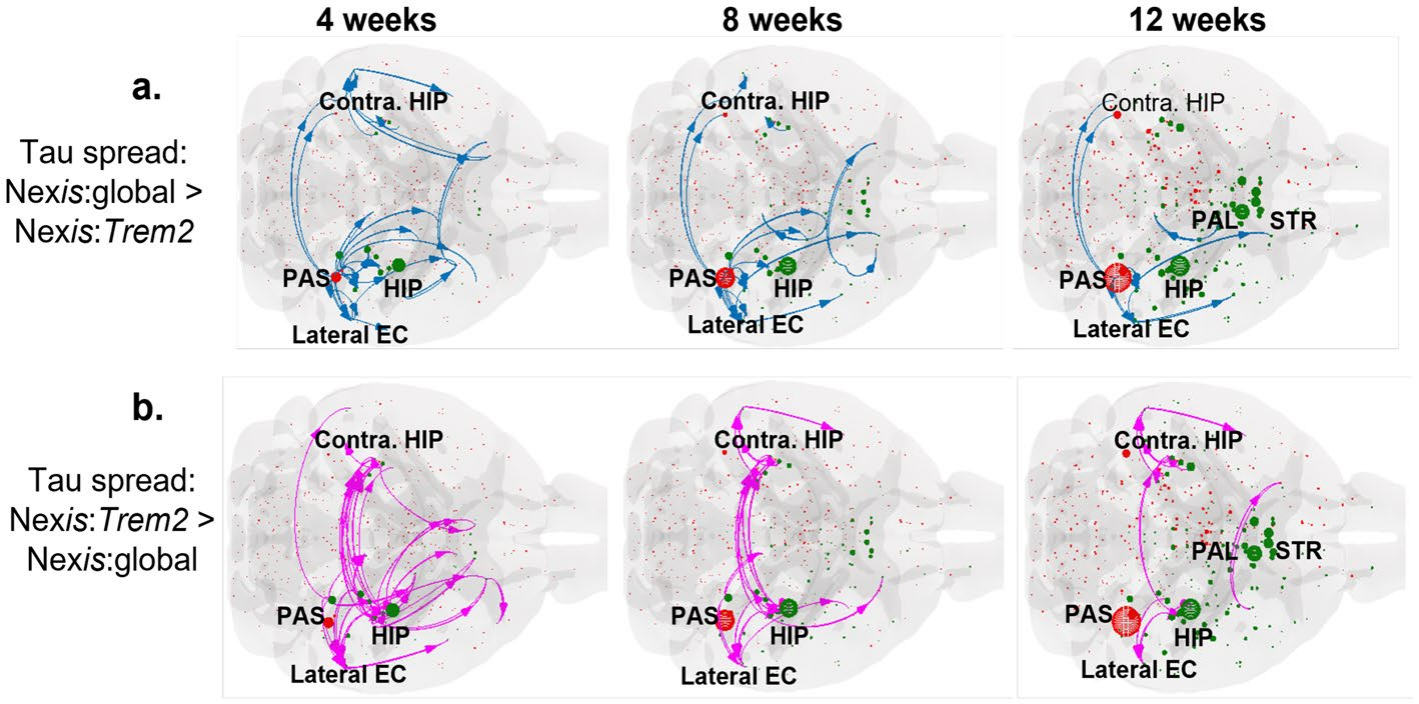
Flow-map representations of tau accumulation and spread in DS9 dataset post hippocampal seeding. Tau burden and spread differences were evaluated between Nex*is*:global and Nex*is*:*Trem2* modeled tau pathology. Blue arrows of tau spread (**a**) depict Nex*is*:global-modeled tau flow without the influence of *Trem2* and indicate a positive difference (tau spread: Nex*is*:global > Nex*is*:*Trem2*). Magenta arrows of tau spread (**b**) depict tau-flow mediated by *Trem2*, above and beyond that modeled by Nex*is*:global and indicate a negative difference (tau spread: Nex*is*:global < Nex*is*:*Trem2*). Red and green spheres represent higher tau burden as modeled by Nex*is*:global and Nex*is*:*Trem2*, respectively. Sphere sizes correspond to the degree of tau burden. HIP: hippocampal area, contra. HIP, contralateral hippocampal area; lateral EC, lateral entorhinal cortex; PAL, pallidal area; PAS, parasubiculum; STR, striatal area.

We next assessed, visually, the effect that microglia are imparting on the spread of tau within the model. Flow-maps depicting major pathology spreading directions were constructed using Brainframe, our in-house Matlab-based function. Figure 5 highlights differences in tau burden and spread as modeled by Nex*is*:global and Nex*is*:*Trem2* for DS9. A prevalence of magenta arrows (Fig. 5b) indicates that *Trem2* allowed more widespread propagation of tau (also reflected in the higher *b* values in Table 1 for DS9). Although modeled tau propagated from the hippocampal seed area to connected regions, *Trem2* appeared to mediate more spread of tau to the striatal, entorhinal, pallidal, and contralateral hippocampal regions. Similarly, a prevalence of green spheres in the striatal, pallidal, and hippocampal areas indicates that these regions evidence higher tau burden due to *Trem2* than would otherwise be expected. Tau burden was unaffected by the presence of *Trem2* in the parasubiculum (red sphere at all time points).

### Changes in *Trem2* expression and the subsequent response of tau

**Figure 6:**
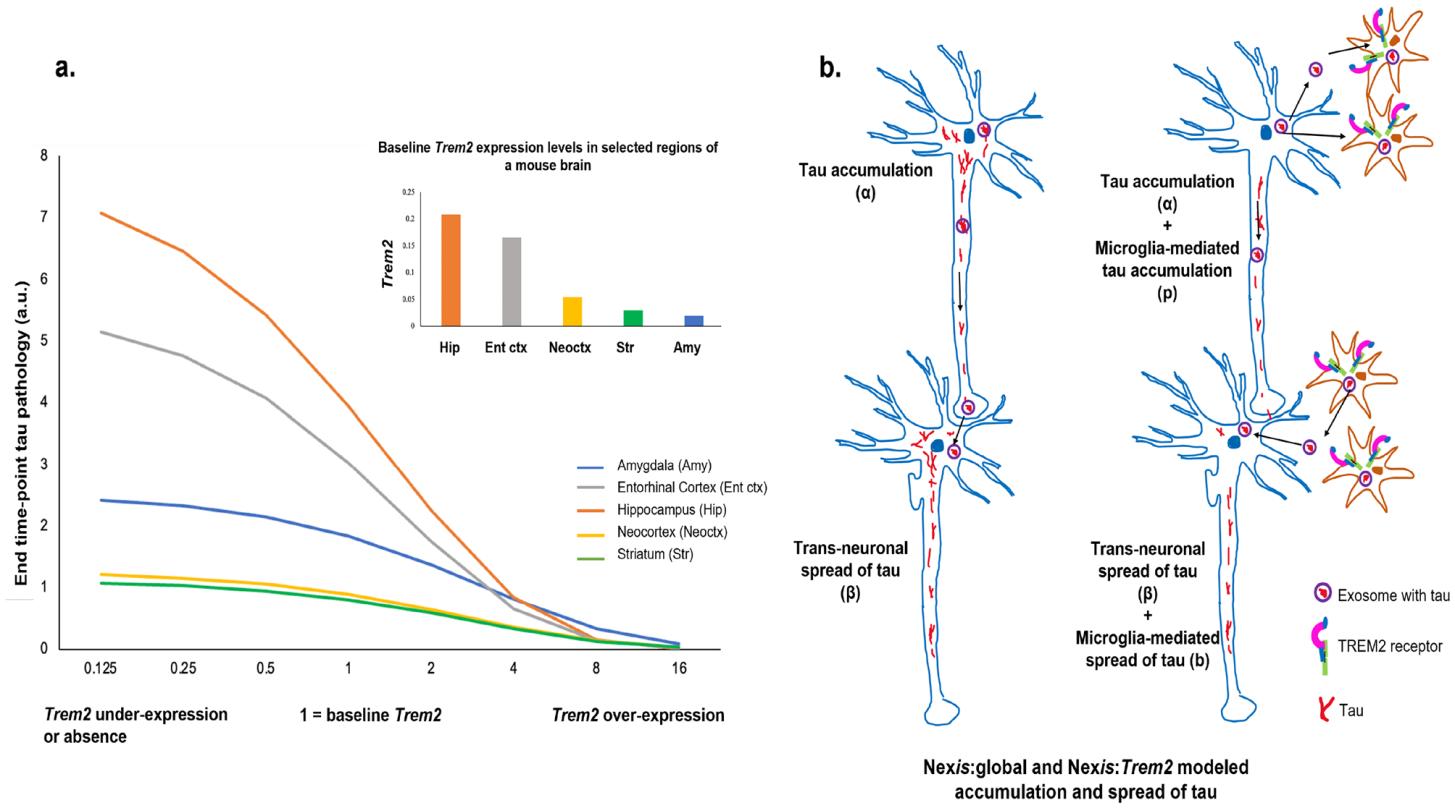
Summary of the relationship between *Trem2* and tau. Panel (**a**): End time-point (12 weeks here) tau levels in response to simulated scaling of *Trem2* expression levels in selected regions of the mouse brain. The inset depicts the baseline expression levels of *Trem2* in the selected brain regions. Panel (**b**): Summary of the effect of *Trem2* on tau accumulation and spread as modeled by Nex*is*:*Trem2*. The rate of tau accumulation (parameter *p*) was lower and the rate of tau spread (parameter *b*) was higher in the presence of *Trem2*. Nex*is*:global, which does not consider the effect of microglia, failed to capture the entire dynamics of tau, leading to poor model performance. Note that the illustrated relationship depends on the strain of tau being modeled. Note also that our speculation of exosomes contributing to trans-neuronal and microglia-mediated spread of tau has not been modeled using Nex*is*.

Given the effect of *Trem2* on model performance and its quantified effects on tau transmissibility (*b*), we hypothesized that there would be a dose-dependent effect of *Trem2* on tau, where higher *Trem2* expression would lead to lower tau levels. *In silico* simulation of changes in baseline *Trem2* levels at the global brain level, followed by application of the Nex*is*:*Trem2* model, indicated a unidirectional trend in tau. Higher *Trem2* expression resulted in a reduction of tau in five sample regions of the mouse brain (Fig. 6a). Specifically, there appeared to be a preponderance of an early effect of *Trem2* in the hippocampus and the entorhinal cortex.

### Nex*is*:microglia models applied to additional candidate genes implicated in tauopathies

We investigated *Apoe*, a strongly implicated AD-risk gene^33–35^ expressed both in microglia and astrocytes^36^. Nex*is*:*Apoe* did not outperform Nex*is*:global in any of the datasets (Table S2, Fig. S4). Since *Trem2* and *Apoe* also work in concert in AD^37^, we conducted a combined Nex*is*:microglia analysis including them both, but results for all three datasets matched those produced by Nex*is*:*Trem2* alone (Table S2). We then applied Nex*is*:microglia to some candidate genes involved in phagocytosis and inflammation (*Sorl1*, *Cd33*, *Clu*, *Sirpa*, and *Aif1*). The accumulation (*p*) and spread (*b*) parameters obtained for these genes were either negative or insignificant, agreeing with their role in containing and eliminating pathological entities.

## Discussion

The spread of hyperphosphorylated tau in AD occurs along the brain’s connectome and is also influenced by the surrounding molecular players such as certain cell types. The presented modeling effort was inspired by and expands upon the “molecular nexopathy” paradigm^5^ in that a central role is noted for network-mediated spread, but also includes other sources of protein aggregation and mediators of trans-neuronal spread, especially microglia. Microglia co-localize with tau inclusions, taking up and releasing bioactive seed-competent tau, and thus emerging as key mediators of AD pathology propagation along with connectome-mediated spread^18,38^. Phagocytic microglia, instead of degrading tau, may package it into exosomes^19^ and form aberrant junctions with surrounding neurons through which tau might be transmitted between cells^18,26^. While prior studies^13–15^ have successfully quantified key aspects of pathology progression along the connectome, they do not constitute a true Nexopathy model, since they did not account for the influence of non-neuronal modulators.

Thus, here we presented a mathematical model called Nex*is*, that attempts to incorporate diverse effects including misfolded protein seeding, aggregation, connectome-mediated spread, as well as the modulation of all the above processes by extra-connectomic agents residing within the brain’s neurological milieu. Nex*is* augments prior quantitative models (NDM)^13^ by including microglial modulation via two key processes: local tau accumulation and its enhanced trans-neuronal transmission. Our results quantify how the inclusion of microglia improves the predictive power of the model and reduces the uncertainty in the accumulation and diffusion parameter estimates (see AIC and narrower confidence intervals around model parameters, Table 1). The full model recapitulates empirical data with $R^2$ values up to 0.60. The NDM, on the other hand, cannot capture the extent of the spatiotemporal pathology because it fails to capture scale. Thus even though the individual Pearson *r* values per time-point are significant, the overall model fit ($R^2$) is very poor with the NDM.

The Nex*is*-modeled spatiotemporal progression of tau closely matches the Braak staging scheme^10^, with pathology progressing from the hippocampal injection site to the contralateral hippocampus and entorhinal cortex, followed by the neocortical, striatal, and thalamic areas. We acknowledge that the data used here were from a seeded mouse model, which may have biased the starting point of pathology to be the hippocampus. Future models applied to unseeded mouse models of AD would help since they do demonstrate tau spatiotemporal behavior as dictated by Braak staging^39^. In addition, the data are from a model of primary tauopathy, and hence cannot necessarily be characterized in the same way as AD tauopathy. This is because there is insufficient information on staging of primary tauopathies^40^, and the classical pattern of tau spread to the frontal regions might be due to the influence of amyloid-$\beta$ in AD.

Microglial modulation was strongly dependent on the tau strain used in the specific dataset. Datasets DS6 and DS9 have medium to high seeding capacity and are characterized by tau from a P301S tauopathy mouse and recombinant fibrils, respectively. For these datasets, *Trem2* was associated with decreased tau accumulation rate (*p*) and increased spread (*b*) to regions ipsi- and contralateral to the hippocampal seed area (Table 1). Moreover, *Trem2* distribution (Fig. 2b) did not spatially correlate with regions with higher *Trem2*-mediated tau burden (Fig. 4d), suggesting that microglial effects are not strictly local. Instead, as shown by differences in fitted model parameters, *Trem2* appears to mobilize tau increasing its network transmissibility. We hypothesize that *Trem2* renders tau more mobile, thus increasing its brain-wide spread but reducing its accumulation rate. Although not possible to model here, the effect of *Trem2* on tau mobilization may be occurring via the action of exosomes^19^. *Trem2*-mediated high tau efflux with slower accumulation may reflect clearance and possible mitigation of pathology. Mouse studies have indeed demonstrated *Trem2*-mediated decrease in tau accumulation in early disease stages^41,42^, *Trem2*-mediated neuroprotection^43^, as well as *Trem2* loss resulting in enhanced tau seeding and exacerbated pathology^24^. Microglia can internalize and release seed-competent tau, but also potentially degrade its seeding activity^38^. Our model parameters here suggest that the decreased accumulation rate (*p*) may support the empirically observed reduced seeding activity^38^, whilst still maintaining tau spreading potential (*b*). Intriguingly, our results posit this effect of microglia to be specific to *Trem2*, and not to homeostatic microglial genes.We schematically summarize the relationship between *Trem2* and tau as modeled by Nex*is* and as observed in these datasets, in Fig. 6.

Neither Nex*is* framework recapitulates one of the datasets, DS4, particularly well (Table 1). DS4, characterized by the human AD-brain derived tau isolate, demonstrated elevated rates of both tau accumulation (*p*) and spread (*b*) in the presence of either *Trem2* or homeostatic microglia. However, neither Nex*is*:homeostasis nor Nex*is*:*Trem2* was significantly better than Nex*is*:global. As observed by Kaufman et al.^27^, DS4 was a low-tangle tau strain with lower seeding capacity than DS6 and DS9. This may explain its comparatively weaker Nex*is*:global model fit, with a failure of any additional improvements with the inclusion of microglial genes. We also speculate that since DS4 was characterized by amyloid-comorbid tau from a human-AD brain it evinced higher values of accumulation and spread rates. AD derived amyloid-comorbid tau can acquire more pathogenic conformations leading to its aggregation and accumulation at a faster rate^44^. Our results thus point to the importance of quantifying the heterogeneous effects that tau has on neuropathology progression, importantly because tau strain diversity based on differences in bioactivity (seeding and spreading) may explain the myriad of AD clinical outcomes^45^.

*Trem2* is upregulated in microglia during disease progression, and in fact a lack of *Trem2* worsens tau pathology^46^. Similar observations have been made by Zhu et al.^47^ where *Trem2* deletion caused enhanced spread of tau pathology from the injection site to other connected regions of a *Trem2* knock-out mouse model. We showed here that Nex*is* is capable of confirming the histopathologically observed mechanistic relationship between *Trem2* and tau (Fig. 6a). *Trem2* expression levels lower and higher than baseline simulated its deletion (or knock-out) and over-expression, respectively. Nex*is*:*Trem2* set up using varying scales of *Trem2* expression could provide quantifiable trends of its inverse relationship with tau as quantified in five sample regions of the mouse brain. The baseline expression levels of *Trem2* (Fig. 6a inset) in these regions is in agreement with the pattern observed in murine and human studies^48^, with the hippocampus and entorhinal cortex evidencing the highest levels followed by the neocortical and striatal regions. *Apoe* is a canonical risk-gene implicated in AD, and along with being a strong influencer of amyloid pathology, it is also suggested to influence microglial function and tau pathology^49^. We thus explored the effects of *Apoe* via Nex*is*:microglia model alone and in conjunction with *Trem2* as these two genes appear to act in concert^37^. Compared to Nex*is*:*Trem2*, the lack of improvement in Nex*is*:*Apoe* and comparable performance by Nex*is*:*Trem2*:*Apoe* can be related to the fact that *Apoe* acts downstream of *Trem2*^37^ (in disease conditions). Therefore, the key mediatory role manifested by *Trem2* can be expected to encompass the contribution of *Apoe*.

In conclusion, our *in silico* Nex*is*:microglia model quantifies the contribution of microglia to tau neuropathology progression. It recapitulates the characteristics of observed tau pathology progression, providing distinct results specific to the tau species employed in the mouse models we studied. Although prior bench studies have suggested a modulatory role for microglia, the present study is the first to demonstrate this *quantitatively* on the whole mouse brain, and for specific microglial genes and specific tau conformations. In particular, our results provide quantitative support to the observations made by Asai et al.^19^ in that, microglia aid increased propagation of tau across brain regions. The Nex*is*:microglia framework can greatly aid experimentalists in further hypothesis generation, validation, and quantification. It may be readily adapted to interrogate the effects of other putative modulators of pathology progression such as astrocytes and oligodendrocytes.

## Limitations and future directions

The datasets used monitored the levels of exogenous tau at 4, 8, and 12 weeks post hippocampal seeding. Additional time-points may enable a more thorough understanding of the spatiotemporal behavior of tau progression. The current monitoring over time was also not longitudinal in the real sense since measurements did not track the same animal. Instead, tau progression was observed in groups of tau-injected mice sacrificed at different time-points post-injection. Further, the gene expression data used as microglial signatures were from a healthy mouse brain and thus can infer only about the effects of baseline microglial density on pathology. However, interestingly, a recent study indeed supports the effect of baseline microglia, rather than the brain’s anatomic network, on tau propagation^26^. We were also restricted to using the coronal series from the AGEA due to its superior spatial coverage relative to the sagittal series, but with significantly fewer quantified genes. As a result, not all microglial genes of interest could be assessed with Nex*is*.

Next, *Trem2* assumes opposing roles (protective vs. detrimental) in AD pathology depending on disease-stage. Such disease-stage dependent dual roles of *Trem2* have been observed with respect to amyloid $\beta$ plaque pathology^50^. Our current *in silico* investigation of the role of *Trem2* in neuropathology progression is restricted to tau and is conducted on data acquired within three months of exogenous tau seeding in mice. Future goals would be to apply this approach to data collected at extended time-points post tau-seeding (depending on data availability), as well as to include amyloid pathology in the model since amyloid $\beta$ has been speculated to potentiate microglial influence on tauopathy propagation^26^. A possibility that the seed region where tau was initially injected may confer specific molecular vulnerability to tau, remains outside the scope of the current study design. Our Nex*is* approach itself is fully capable of testing for site-specific effects but paucity of experimental data to date makes this challenging.

We also note that the nexopathy paradigm enumerates a far more diverse set of potential mechanisms by which molecular dysfunction might interact with the neural architecture than is possible to explore in a single study. Epigenetic^51^ or post-translational modifications^52^ due to variable causes will produce gene expression in diseased groups that is different from healthy gene expression used here. However, the primary goal of uncovering the molecular versus network correlates of AD topography may be largely unaffected by these additional factors, since they are either unlikely to have spatial gradients (e.g. age, environment) or have non-AD-like spatial patterning (oxidative/vascular stress).

Finally, the current modeling attempts were restricted to data from mouse models of primary tauopathy. There are two main reasons why we decided to start with mouse models: (1) the datasets compared here are from mice having the same genetic background thus minimizing any effects of genetic heterogeneity on model results, and (2) since mice are exogenously injected with pathology in specific brain regions, pathology progression from a common seed location can be modeled and compared. Pathology seeding data from humans are not possible, and despite the stereotyped progression of AD tau pathology, there is evidence that significant heterogeneity exists between patients at disease inception^53^. Another advantage of seeded mouse models is that the diverse effects of different strains of the pathological species can be compared. That being said, the true translational aspect of Nex*is* will be appreciated by its application to human data. This will also be important for inter-species comparison of the glial response to pathology, since transcriptional signatures of glia in AD pathology can be markedly different between mouse AD models and specimens from human AD patients^54^. Nevertheless, despite this difference, the fact that *Trem2* impacts microglia function is a common theme in mouse and human AD^54^. We are currently in the process of curating and inspecting human data to which we can apply Nex*is*.

## Methods

### Study design

Empirical spatiotemporal tau pathology data were obtained from a study on PS19 mice expressing 4R1N P301S with human tau, exogenously injected with distinct strains of pathological tau^27^. Regional levels of tau burden were extracted from heat maps displaying semi-quantified pathology anatomically following the approach from Mezias et al.^55^. The Kaufman datasets used in this study are DS4 (containing tau isolate derived from the human AD-brain), DS6 (contained tau injectate from a P301S tauopathy mouse), and DS9 (characterized by recombinant tau fibrils). Our 426-region mouse connectome is from the Allen Brain Atlas (ABA)^28^, with 213 region bi-hemispheric symmetry. The microglial baseline spatial gene expression maps employed as signatures of baseline homeostatic and phagocytic microglia are from the ABA gene expression atlas (AGEA) of healthy 56-day old male C57BL/6J mice^30^. Note that these microglial signatures represent quiescent rather than activated states and may not be considered disease-activated microglia (DAM). Moreover we selectively use only the genes from the coronal AGEA and exclude those from the sagittal atlas because the spatial coverage of gene expression is higher in the former. Thus, the final gene list that we referred to has a subset containing 3855 genes from a total of approximately 26000 genes in the AGEA (sagittal + coronal).

Regional homeostatic microglial abundance was defined as the first principal component of the expression levels of *P2ry12*, *Cx3cr1*, *Fcrls*, *Olfml3*, *Hexb*, *Siglech*, *Sox5*, and *Jun*^29,56^. We separately tested the influence of *Trem2*, *Cd33*, and *Apoe*, activated microglial surface-markers and regulators of microglia-mediated phagocytosis strongly implicated in AD pathogenesis^57^. *Cd33*’s influence (Table S3) was negligible compared to *Trem2* given its smaller effect size compared to *Trem2*^58^.

### Nexopathy *in silico* Model (Nex*is*)

The Nex*is* model is derived from the Network Diffusion Model (NDM)^13^—a mathematical model that describes pathology spread as a diffusive process between connected brain regions. Let $c_{12}$ denote the inter-region connection strength between two brain regions (1,2) as inferred from tractography data, let and $x_{1}(t)$ and $x_{2}(t)$ be the pathology concentration at two brain regions at time *t* as inferred from semi-quantitative immunohistochemistry analysis from the specific reference study. Following the NDM^13^, we impose first-order diffusive dynamics:

1 $$\begin{aligned} \frac{dx_{1}}{dt}= \beta c_{12} (x_{2}-x_{1}), \end{aligned}$$

where $\beta$ is the global diffusivity rate that controls how fast pathology can flow from the more concentrated region to the less concentrated one independent of any other modulators.

**Nex*is***:**global** adds another parameter to the NDM, $\alpha x_{1}$, a linear accumulation or clearance term depending on its sign.

2 $$\begin{aligned} \frac{dx_{1}}{dt}= \beta c_{12} (x_{2}-x_{1}) + \alpha x_{1}, \end{aligned}$$

This pair-wise relationship can be extended to the entire brain connectivity network using vector $\vec{x} \in {\mathbb {R}}^{n}$ over all *n* ROIs, to give $\frac{d\vec{x}}{dt} = -\beta L \vec{x}$, where *L* is the graph Laplacian matrix (when $\alpha = 0$)^13^.

### Nex*is*:microglia

The Nex*is*:microglia framework incorporates the potential effects that different modulators, for instance cell-types such as microglia, may have on neuropathology progression, particularly in the context of AD and other tauopathies. It has two additional cell-type specific parameters *p* and *b*, which indicate the effect of the chosen cell type, *u*, on pathology accumulation/clearance and spread, respectively. Letting $u_{1}$ and $u_{2}$ indicate the local gene expression in regions 1 and 2, the extended pairwise relationship in (2) becomes

3 $$\begin{aligned} \frac{dx_{1}}{dt} = \beta c_{12} \big [(1 + u_{2}b)x_{2} - (1 + u_{1}b)x_{1}\big ] + \alpha x_{1} + u_{1} p x_{1}. \end{aligned}$$

Equation (3) reduces to NDM equation (1) if $b = p = \alpha = 0$, and can similarly be extended brain-wide via pathology $\vec{x}$ and gene $\vec{u}$ vectors to $\frac{d \vec{x}}{dt} = [\Lambda ( \vec{u} ) - \beta {\tilde{L}}( \vec{u} )] \vec{x}$, where $\Lambda$ is a diagonal matrix and ${\tilde{L}}$ is a new Laplacian, both now depending on gene expression *u*. See the Supplementary Information for a detailed explanation.

For convenience of reading, in the remainder of the document the model name ’Nex*is*:microglia’ will be replaced by Nex*is*:homeostasis in the case of homeostatic microglia or Nex*is*:(*microglial gene name*) in the case of a particular microglial gene being investigated.

### Seed scaling and model initialization

Both NDM and Nex*is* require a seed-rescale parameter $\gamma$, which denotes the scaling factor for initial pathology at the site of injection of pathology (tau in the current case). This parameter needs to be quantified since, although we know the site of the injection (hippocampus for instance), we do not know the amount of pathology being injected. Thus, the pathology at *t* = 0 is represented as a binary vector multiplied by $\gamma$ and is used to initialize the model.

### Robust strategy for parameter estimation

We implemented a two-step, robust strategy for estimating parameters for the NDM and Nex*is* models. First, we fit the spread parameter ($\beta$) for the NDM, followed by the accumulation and spread parameters ($\alpha$ and $\beta$) for Nex*is*:global via grid search and random initialization. This was then followed by 100 iterations of bootstrapping with 80$\%$ re-sampling. Next, for the Nex*is*:microglia model, we initialized the values of $\alpha$ and $\beta$ to those obtained from Nex*is*:global while also fitting (without initialization) the microglia-mediated accumulation and spread parameters (*p* and *b*). Anchoring the parameters shared by both Nex*is* frameworks within a reasonable range from each in the optimization routines prevents the algorithm from reaching local minima that yield better fits but have overall biologically-implausible parameter values. This also allowed us to assess reproducibility by acquiring the 95% confidence intervals around the mean estimates of the model fit as well as global and microglial parameters (simulations in Fig. 3 for Nex*is*:global and Nex*is*:*Trem2*). Importantly, model parameters were fit using all available quantified regions and all time points; prior formulations only allowed fitting to single time points^14,15^.

### Statistics and reproducibility

The statistical significance of the differences in model fits between the NDM and the Nex*is* frameworks was determined by carrying out Fisher’s-R-to-Z transformation. The *p* value of significance was obtained by the Student’s t test on the Fisher-transformed Z scores. The model fits presented in Table 1 were determined by averaging the parameters across 100 iterations of bootstrapping with 80% re-sampling of the regions quantified by Kaufman et al.^27^. When fitting the Nex*is*:microglia models we used the mean and 95% confidence intervals determined for the Nex*is*:global parameters $\alpha$, $\beta$, and $\gamma$ for each dataset as the initial guesses and bounds for those parameters, respectively; this helps to minimize the risk of finding degenerate solutions. Please see Supplementary Information for details on parameter fitting. The $R^2$ values of the full model are within the range of the bootstrapped iterations of the 80% re-sampled data (Fig. 3c), thus confirming reproducibility. All analyses were conducted in Matlab R2020b.

### Simulation of changes in *Trem2* expression to assess response of tau

Significant improvement in model fit upon inclusion of *Trem2* warranted further *in silico* quantification of tau response to simulated changes in *Trem2* expression levels. Baseline *Trem2* levels were scaled by factors of two above and below the baseline level and tau was quantified in the hippocampus, entorhinal cortex, neocortex, striatum, and amygdala. The scaling was performed by multiplying the microglial gene (here, *Trem2*) vector by powers of 2 (from $2^{-3}$ to $2^4$) before entering it into the model.

### Ethics approval and consent to participate

All data were obtained from publicly available datasets and published papers. No human subjects, live animals, patient or animal derived tissue, cell lines, or biological material were directly used in this research and thus, no consent or ethics approval was required.

## Supplementary Information

Supplementary Information.

## Data Availability

The source data on the mouse models, gene expression, and mesoscale mouse connectome as well as the computer code to run the models are available at our repository at https://github.com/Raj-Lab-UCSF/Nexis.
