# Supplementary material for: The effects of microglia on tauopathy progression can be quantified using Nexopathy in silico (Nexis) models
Source: Sci Rep. 2022 Dec 7;12:21170. doi: 10.1038/s41598-022-25131-3 (PMC9729195; doi:10.1038/s41598-022-25131-3)
Supplement: Supplementary file 1 — Supplementary Information. [file 41598_2022_25131_MOESM1_ESM.pdf]

Supplementary Information

The effects of microglia on tauopathy progression can be quantified using Nexopathy *in silico* (Nex*is*) models

Chaitali Anand[1,*,+], Pedro Maia[2,+], Justin Torok[1], Christopher Mezias[3], Ashish Raj[1]

1. Department of Radiology, University of California, San Francisco, CA, USA
2. Department of Mathematics, University of Texas, Arlington, TX, USA
3. Department of Neuroscience, Cold Spring Harbor Laboratory, NY, USA

*: corresponding authors (chaitali.anand@ucsf.edu, ashish.raj@ucsf.edu)

[+]: equal contribution

Contents

1 Overview **2**

2 Model Development **2**

2.1 Description of parameters and notation 2

2.2 Derivation of general model in matrix form 3

3 Cost functions and fitting parameter to data: **4**

4 Strategies for parameter consistency **4**

5 Pseudocode for Nex*is*:global and Nex*is*:microglia **4**

6 Gene selection for Nex*is*:microglia **4**

7 Model parameters and fit for *Apoe* **4**

8 Nex*is* Algorithms **5**

9 Figures **7**

10 Tables **11**

1 Overview

Our previous Network Diffusion Model (NDM) can be extended to incorporate the effects of pathology accumulation/clearance rate in addition to pathology diffusion rate. We call this model Nexopathy *in silico* or Nex*is*. This can be further extended to include the potential effects that different cell-types, such as microglia, may have on the progression of neuropathology, particularly in the context of Alzheimer's disease (AD) and other tauopathies. We thus call this model Nex*is*:microglia, as opposed to Nex*is*:global that is posited without the effects of other cellular and molecular mediators of pathology spread. In particular, Nex*is*:microglia seeks to model two different effects of microglia: 1) modulation of intra-regional accumulation/clearance rate, given by the term M_α and 2) modulation of inter-regional spread rate, given by the term M_β. These hypotheses are not mutually exclusive, but they have different biological and mathematical implications that we explore below.

2 Model Development

2.1 Description of parameters and notation

- t: time
- $x_j(t)$: amount of pathology in brain region j (known)
- c_{ij}: connectivity between regions i and j (known)
- u_{jk}: cell-type density/spatial gene expression k at region j (known)
- α: global amplification/depletion term (unknown)
- β: global diffusivity constant (unknown)
- γ: scaling factor for initial pathology i.e. the amount of exogenous tau injected at the seeding site (unknown)
- b_k: parameter on how $\vec{u}_k$ affects transmission (unknown)
- p_k: interaction parameter between cell type k and pathology (unknown)

M_α: Initiation/Clearance terms

- (i): $\alpha \cdot x_j$ (independent of cell type)
- (ii): $\sum_{k=1}^{n} p_k \cdot u_{jk} \cdot x_j$ (cross-terms between x_j and u_j)

M_β: Inter-regional spread terms

- (i): $\beta \cdot \sum_i [c_{ij}x_i - c_{ji}x_j]$ (Nex*is*:global)
- (ii): $\beta \cdot \sum_i [c_{ij}(1+\sum_k b_k u_{ik})x_i - c_{ji}(1+\sum_k b_k u_{jk})x_j]$ (Nex*is*:microglia, re-weighted by cell types)

We denote vectors with arrows and matrices with capital letters. The Nex*is*:microglia equation takes the form

$$\frac{dx_j}{dt} = \underbrace{M_\alpha(\vec{x}|U,\alpha;\vec{a},\vec{p}),}_{\text{accumulation/clearance}} + \underbrace{M_\beta(\vec{x}|C,U,\beta,\vec{b})}_{\text{inter-regional spread}} \tag{1}$$

The vector $\vec{x} \in \mathbb{R}^n$ denotes the pathology measured along n regions of interest (ROIs), with an entry x_j denoting the pathology at region j. The vectors $\vec{u}_1, \vec{u}_2, \ldots, \vec{u}_k$ denote the cell-type densities along the same n ROIs and are stored in the matrix U.

$$U = \begin{bmatrix} | & | & & | \\ \vec{u}_1 & \vec{u}_2 & \cdots & \vec{u}_k \\ | & | & & | \end{bmatrix}$$

The parameters $\{\alpha;\vec{a},\vec{p}\}$ are associated with initiation/clearance of pathology and $\{C,U,\beta,\vec{b}\}$ are associated with inter-regional spread.

2.2 Derivation of general model in matrix form

We begin by writing all terms explicitly for (i)-(ii) for M_α and (ii) for M_β on a system with 3 ROI given by x_1, x_2, and x_3. We also define the following auxiliary variables:

$$\begin{aligned} s_j^b &= \sum_k u_{jk} \cdot b_k \\ s_j^p &= \sum_k u_{jk} \cdot p_k \end{aligned}$$

The model then writes:

$$\begin{aligned} \dot{x_1} &= \beta\left[c_{21}(1+s_2^b)x_2 - c_{12}(1+s_1^b)x_1 + c_{31}(1+s_3^b)x_3 - c_{13}(1+s_1^b)x_1\right] + \alpha x_1 + s_1^p x_1 \\ \dot{x_2} &= \beta\left[c_{12}(1+s_1^b)x_1 - c_{21}(1+s_2^b)x_2 + c_{32}(1+s_3^b)x_3 - c_{23}(1+s_2^b)x_2\right] + \alpha x_2 + s_2^p x_2 \\ \dot{x_3} &= \beta\left[c_{13}(1+s_1^b)x_1 - c_{31}(1+s_3^b)x_3 + c_{23}(1+s_2^b)x_2 - c_{32}(1+s_3^b)x_3\right] + \alpha x_3 + s_3^p x_3 \end{aligned}$$

Rearranging terms and grouping them as factors of x_1, x_2 and x_3 yields

$$\begin{aligned} \dot{x_1} &= [\alpha + s_1^p - \beta(c_{12}+c_{13})(1+s_1^b)]x_1 + [\beta c_{21}(1+s_2^b)]x_2 + [\beta c_{31}(1+s_3^b)]x_3 \\ \dot{x_2} &= [\beta c_{12}(1+s_1^b)]x_1 + [\alpha + s_2^p - \beta(c_{21}+c_{23})(1+s_1^b)]x_2 + [\beta c_{32}(1+s_3^b)]x_3 \\ \dot{x_3} &= [\beta c_{13}(1+s_1^b)]x_1 + [\beta c_{23}(1+s_2^b)]x_2 + [\alpha + s_3^p - \beta(c_{31}+c_{32})(1+s_3^b)]x_3 \end{aligned}$$

The terms of the RHS of the system of equations can be expressed as a matrix-vector multiplication. Note that the diagonal elements of the matrix have a term of the form $\alpha + s_j^p$. For convenience, we will incorporate them in a diagonal matrix Λ, that easily generalizes for n regions as shown below:

$$\begin{bmatrix} \Lambda_{11} & 0 & 0 & \cdots & 0 \\ 0 & \Lambda_{22} & 0 & \cdots & 0 \\ 0 & 0 & \Lambda_{33} & \cdots & 0 \\ \vdots & \vdots & \vdots & \ddots & \vdots \\ 0 & 0 & 0 & \cdots & \Lambda_{nn} \end{bmatrix}, \text{where } \Lambda_{jj} = \alpha + s_j^p.$$

To be consistent with previous studies, we will write the remaining matrix as $-\beta L = -\beta(D-C)$, where D is a diagonal matrix. The off-diagonal terms take a simpler form, $L_{ij} = -c_{ij}(1+s_i^b)$, that easily generalizes for n dimensions. The diagonal terms, however, must be augmented to include $c_{j4} + \cdots + c_{jN}$. In these sums, only the term c_{jj} is missing in line j. Thus, to write the diagonal terms in a more compact form, we introduce the following trick:

$$r_j = -c_{jj} + \sum_i c_{ji}.$$

The RHS of the general model in matrix form becomes

$$\left(\begin{bmatrix} \Lambda_{11} & 0 & 0 & \cdots & 0 \\ 0 & \Lambda_{22} & 0 & \cdots & 0 \\ 0 & 0 & \Lambda_{33} & \cdots & 0 \\ \vdots & \vdots & \vdots & \ddots & \vdots \\ 0 & 0 & 0 & \cdots & \Lambda_{nn} \end{bmatrix} - \beta \begin{bmatrix} L_{11} & L_{12} & L_{13} & \cdots & L_{1n} \\ L_{21} & L_{22} & L_{23} & \cdots & L_{2n} \\ L_{31} & L_{32} & L_{33} & \cdots & L_{3n} \\ \vdots & \vdots & \vdots & \ddots & \vdots \\ L_{n1} & L_{n2} & L_{n3} & \cdots & L_{nn} \end{bmatrix} \right) \begin{bmatrix} x_1 \\ x_2 \\ x_3 \\ \vdots \\ x_n \end{bmatrix} \tag{2}$$

where

$$\begin{aligned} \Lambda_{jj} &= \alpha + s_j^p \\ L_{jj} &= (1+s_j^b)r_j \\ L_{ij} &= -c_{ij}(1+s_i^b), \text{ when } i \neq j \end{aligned}$$

With the definitions above, the model reads:

$$\frac{d\vec{x}}{dt} = [\Lambda - \beta L]\vec{x} \tag{3}$$

If all $b_k = 0$ or if all $u_{jk} = 0$, $s_j^b = 0$ for all j and the L matrix will reduce to the familiar (unnormalized) Laplacian matrix. It is also worth noting that the degrees of freedom of this system is equal to $3k+2$, which grows unwieldy when the total number of cell types, m, is large given the low temporal resolution of most pathology data.

3 Cost functions and fitting parameter to data:

From equation (3), we can predict the amount of pathology $\vec{x}$ at any given time t for a given set of parameters Θ. This prediction can be done either numerically (using MATLAB's ODE solvers) or analytically (if $\vec{s}_a = \vec{0}$, then the ODEs can be solved via matrix exponentiation). In practice, we typically have observed data $\vec{y}$ at times $t_1, \ldots, t_N$ and we try to find optimal parameters Θ that will minimize the discrepancies between $\vec{y}(t_i)$ and $\vec{x}(t_i)$. Our Nex*is*:microglia code provides multiple choices of cost functions: (a) Mean Square Error, (b) Mean Absolute Error, (c) Pearson R, and (d) Lin R. For the particular datasets used in this study, the Lin R cost function provided the overall best solutions, although this may vary from problem to problem. The vector of initial pathology, $\mathbf{x_0}$, also merits further explanation. For all studies, we create a binary vector, $\mathbf{x}_{\text{bin}}$, which either indicates the regions in which pathology was injected or was present at the earliest time point. Since the scale of the pathology at the time of injection is unknown and is unrelated to the scale of $\mathbf{x}_{\text{bin}}$, we introduce a parameter, γ, to be fit from the following relation:

$$\mathbf{x_0} = \gamma \cdot \mathbf{x}_{\text{bin}} .$$

4 Strategies for parameter consistency

The user should provide plausible lower and upper bounds for all parameters in the model. For instance, the user may first optimize all parameters for the Nex*is*:global model followed by determining the fit of the model after 100 iterations of bootstrapping with 80% resampling of the regions quantified by the study being used. For fitting the Nex*is*:microglia, the mean and 95% confidence intervals determined for the Nex*is*:global parameters α, β, and γ are used as the initial guess and bounds for those parameters, respectively.

5 Pseudocode for Nex*is*:global and Nex*is*:microglia

All model fits presented in the current study were determined by averaging the parameters across 100 iterations of bootstrapping with 80% resampling of the regions quantified by Kaufman, *et al.* When fitting the Nex*is*:microglia model, we used the mean and 95% confidence intervals determined for the Nex*is*:global model parameters α, β, and γ for each dataset as the initial guess and bounds for those parameters, respectively; this helps to minimize the risk of finding degenerate solutions. We describe the procedure more fully in Algorithms 1 and 2 below.

6 Gene selection for Nex*is*:microglia

Based on literature survey we selected eight microglial homeostasis genes (*P2ry12*, *Cx3cr1*, *Fcrls*, *Olfml3*, *Hexb*, *Siglech*, *Sox5*, and *Jun*) as well as *Trem2*, a surface-marker of activated microglia. For convenience of reading, in the remainder of the document, the model name 'Nex*is*:microglia' will be replaced by Nex*is*:homeostasis in the case of homeostatic microglia or Nex*is*:(*microglial gene name*) in the case of a particular microglial gene being investigated.

The Nex*is*:microglia models were constructed using either the individual microglial gene *Trem2* or the first principal component of only the microglial homeostatic signature genes. We then compared model fits of the Nex*is*:global to those of the Nex*is*:microglia models using the following metrics: AIC, BIC, and Pearson R-squared of observed and predicted pathology spread collapsed across time-points. We also explored the effect of *Apoe* on the model parameters, since *Apoe*, although not exclusively microglial, is an important AD-risk gene. Since the performance of the model with *Apoe* was worse compared to Nex*is*:*Trem2* and comparable to the Nex*is*:global model fit, we do not include its results in the main manuscript but provide them in this SI instead. Table S1 presents model results of separate Nex*is*:microglia models applied to the homeostatic markers. Nex*is*:microglia was applied to some additional candidate genes (*Sorl1*, *Clu*, *Cd33*, *Sirpa*, and *Aif1*) involved in the the microglial phagocytosis and inflammation response (Table S3).

7 Model parameters and fit for *Apoe*

We followed the same strategy of Nex*is*:microglia model fitting and model comparison by using *Apoe* as the target microglial gene. Table S2 includes model parameters and fit indices for Nex*is*:global, Nex*is*:*Trem2*, Nex*is*:*Apoe*, and Nex*is*:*Trem2*:*Apoe* for all three datasets. Figure S4 depicts scatter-plots of observed versus modeled pathology using *Apoe* as well as bootstrapped parameters of the Nex*is*:*Apoe* model for dataset DS9.

8 Nex*is* Algorithms

Algorithm 1: Nex*is*:global Parameter Fitting

Data: $\mathbf{x_i}$, regional tau pathology for dataset i

Result: $\{\alpha^*_{\text{Nex}is\text{:global}}, \beta^*_{\text{Nex}is\text{:global}}, \gamma^*_{\text{Nex}is\text{:global}}\}$, the optimal set of Nex*is*:global parameters, and $\tilde{\mathbf{x}}_\mathbf{i}^{\text{Nex}is\text{:global}}$, the predicted tau pathology for dataset i under the Nex*is*:global.

Function `Nexis:global` $(\mathbf{x_{seed}}, t, \alpha, \beta, \gamma)$ **:**

  $\tilde{\mathbf{x}}(t) \leftarrow$ **solve** Equation [1] with $t = t$, $\alpha = \alpha$, $\beta = \beta$, $\gamma = \gamma$, $b = 0$, $p = 0$, $\mathbf{x_0} = \gamma \cdot \mathbf{x_{seed}}$;

  return $\tilde{\mathbf{x}}(t)$;

End Function;

$\alpha_0 \leftarrow 0.5$;

$\beta_0 \leftarrow 1$;

$\gamma_0 \leftarrow \frac{\sum \mathbf{y_i}(t_1)}{n_{seed}}$;

$\mathbf{x_{seed}} \leftarrow$ binary seed vector for dataset i;

$M_i \leftarrow$ the set of quantified regions in dataset i;

$j \leftarrow 1$;

while $j \leq 100$ **do**

  $M_i^j \leftarrow$ the jth random subset of M_i with cardinality $0.8 \times |M_i|$;

  $\mathbf{x_i^j} \leftarrow \mathbf{x_i}|_{M_i^j}$;

  $\alpha^j, \beta^j, \gamma^j \leftarrow \underset{\alpha,\beta,\gamma}{\text{argmin}} \sum_t (1 - R_C(\mathbf{x_i^j}(t), \text{Nex}is\text{:global}(\mathbf{x_{seed}}, t, \alpha, \beta, \gamma))$ initialized at $\alpha = \alpha_0$, $\beta = \beta_0$, $\gamma = \gamma_0$;

  $j = j + 1$;

end

$\alpha^*_{\text{Nex}is\text{:global}} \leftarrow \sum_j \alpha^j / 100$;

$\beta^*_{\text{Nex}is\text{:global}} \leftarrow \sum_j \beta^j / 100$;

$\gamma^*_{\text{Nex}is\text{:global}} \leftarrow \sum_j \gamma^j / 100$;

$\tilde{\mathbf{x}}_\mathbf{i}^{\text{Nex}is\text{:global}} \leftarrow \text{Nex}is\text{:global}(\mathbf{x_{seed}}, t, \alpha^*_{\text{Nex}is\text{:global}}, \beta^*_{\text{Nex}is\text{:global}}, \gamma^*_{\text{Nex}is\text{:global}})$;

Here $R_C(x,y)$ is the Lin's concordance correlation coefficient between vectors x and y. We find that our heuristic choice of initial α, β, and γ does not impact the results of Algorithm 2.

Algorithm 2: Nex*is*:microglia Parameter Fitting

Data: $\mathbf{x_i}$, regional tau pathology for dataset i

Result: $\{\alpha^*_{\text{Nex}is\text{:microglia}}, \beta^*_{\text{Nex}is\text{:microglia}}, \gamma^*_{\text{Nex}is\text{:microglia}}\}$, the optimal set of Nex*is*:microglia parameters, and $\mathbf{\tilde{x}_i}^{\text{Nex}is\text{:microglia}}$, the predicted tau pathology for dataset i under the Nex*is*:microglia.

Function `Nexis:microglia`($\mathbf{x_{seed}}, t, \alpha, \beta, \gamma$) **:**

  $\mathbf{\tilde{x}}(t) \leftarrow$ **solve** Equation [1] with $t = t$, $\alpha = \alpha$, $\beta = \beta$, $\gamma = \gamma$, $b = b$, $p = p$, $\mathbf{x_0} = \gamma \cdot \mathbf{x_{seed}}$;

  return $\mathbf{\tilde{x}}(t)$;

End Function;

$\alpha_0 \leftarrow 0.5$;

$\beta_0 \leftarrow 1$;

$\gamma_0 \leftarrow \frac{\sum \mathbf{y_i}(t_1)}{n_{seed}}$;

$\mathbf{x_{seed}} \leftarrow$ binary seed vector for dataset i;

$M_i \leftarrow$ the set of quantified regions in dataset i;

$j \leftarrow 1$;

while $j \leq 100$ **do**

  $M_i^j \leftarrow$ the jth random subset of M_i with cardinality $0.8 \times |M_i|$;

  $\mathbf{x_i^j} \leftarrow \mathbf{x_i}|_{M_i^j}$;

  $\alpha^j, \beta^j, \gamma^j \leftarrow \underset{\alpha,\beta,\gamma}{\operatorname{argmin}} \sum_t (1 - R_C(\mathbf{x_i^j}(t));$

  $j = j + 1$;

end

Algorithm 1 is called to obtain initial guesses for α, β, and γ

$\alpha^*_{\text{Nex}is\text{:microglia}} \leftarrow \sum_j \alpha^j / 100$;

$\beta^*_{\text{Nex}is\text{:microglia}} \leftarrow \sum_j \beta^j / 100$;

$\gamma^*_{\text{Nex}is\text{:microglia}} \leftarrow \sum_j \gamma^j / 100$;

$\mathbf{\tilde{x}_i}^{\text{Nex}is\text{:microglia}} \leftarrow \text{Nex}is\text{:microglia}(\mathbf{x_{seed}}, t, \alpha^*_{\text{Nex}is\text{:microglia}}, \beta^*_{\text{Nex}is\text{:microglia}}, \gamma^*_{\text{Nex}is\text{:microglia}})$;

9 Figures

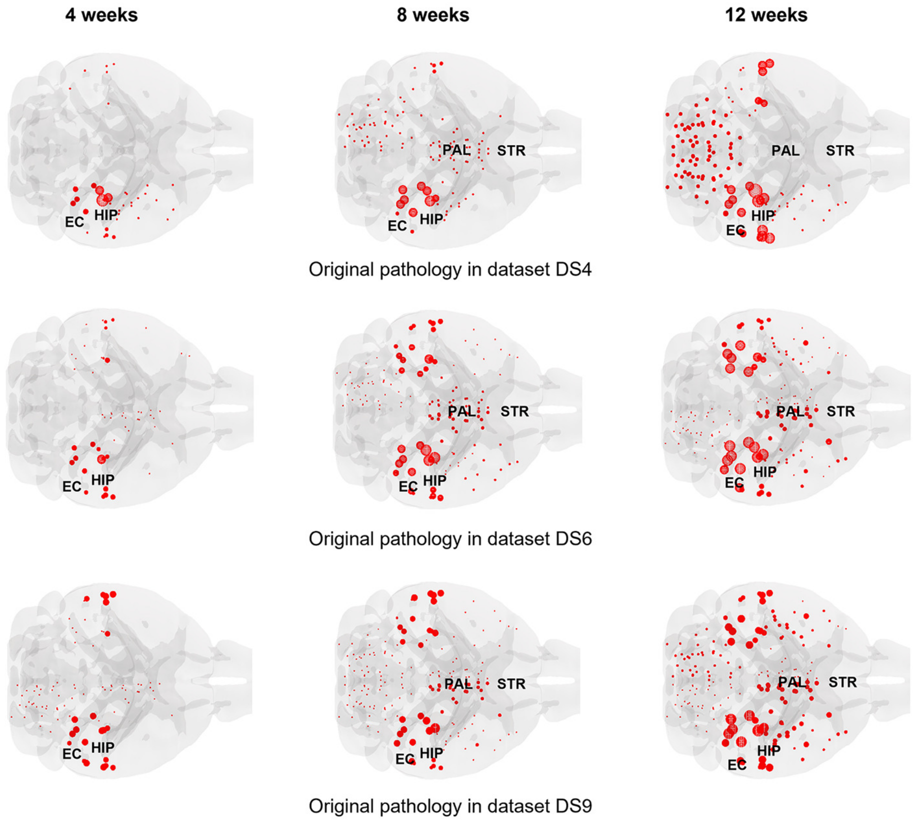

Figure S1. Distinct spatiotemporal pathology in datasets DS4, DS6, and DS9. The varying capacities of tau accumulation and spreading in three datasets as observed in the Kaufman *et al* study have been captured *in silico* for display. Sphere sizes correspond to the degree of tau burden in specific brain regions. DS4 was characterized by medium-level seeding activity and did not show tau pathology in the contralateral regions until 12 weeks after injection. DS6 and DS9 were characterized by the highest seeding activity and pathology induced by these strains progressed to the contralateral side as early as 4 weeks post-injection. (HIP: hippocampus, EC: entorhinal cortex, PAL: pallidum, STR: striatum.)

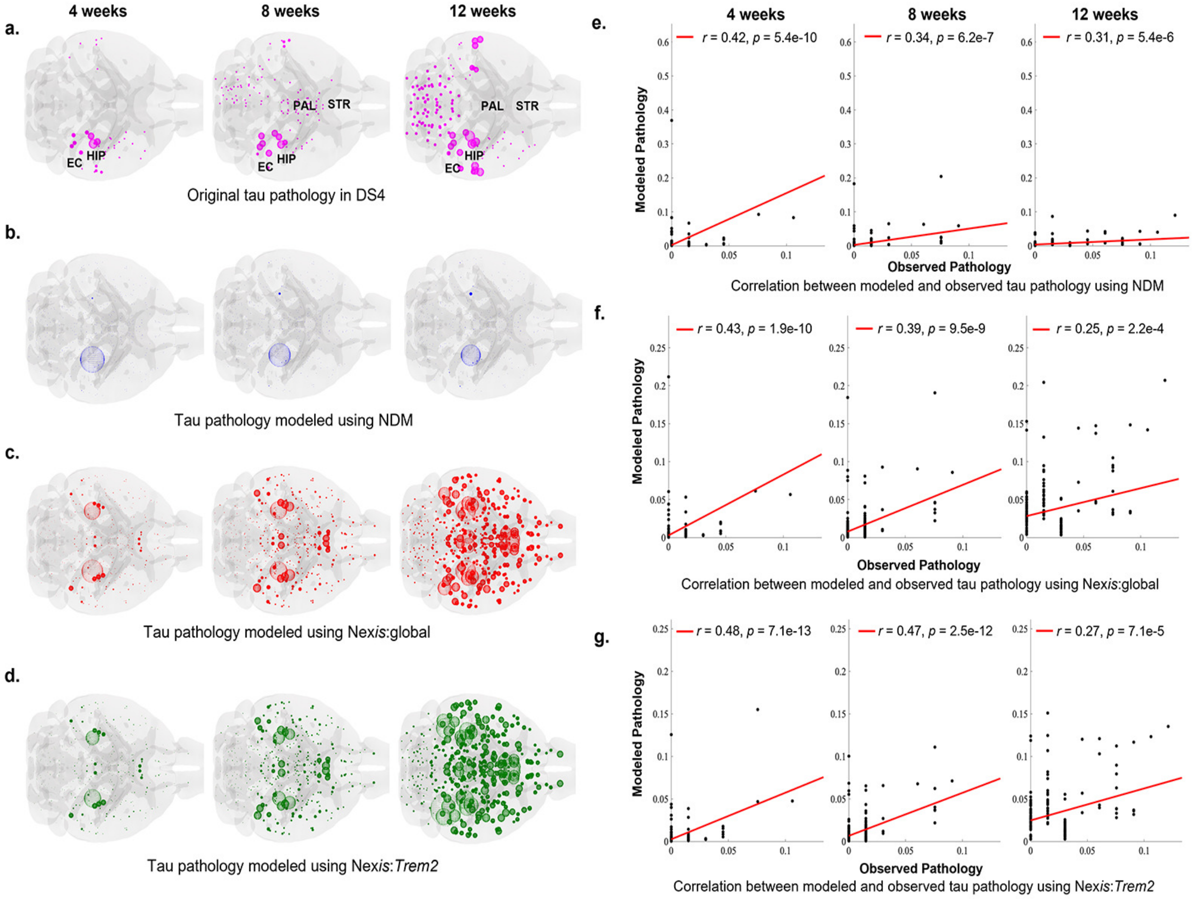

Figure S2. **NDM, Nex*is*:global, and Nex*is*:*Trem2* model fits for dataset DS4.** Panel a depicts empirical tau pathology measured by Kaufman *et al* in dataset DS4. Panels b, c, and d depict NDM, Nex*is*:global, and Nex*is*:*Trem2* modeled tau pathology progression, respectively, in DS4 at 4, 8, and 12 weeks post hippocampal injection. Panels e, f, and g are scatter-plots depicting the correlation between empirical and ND-modeled (e), Nex*is*:global-modeled (f), as well as Nex*is*:*Trem2*-modeled (g) tau pathology, respectively, at 4, 8, and 12 weeks post hippocampal tau seeding. (HIP: hippocampal area, EC: entorhinal cortex, PAL: pallidum, STR: striatum.)

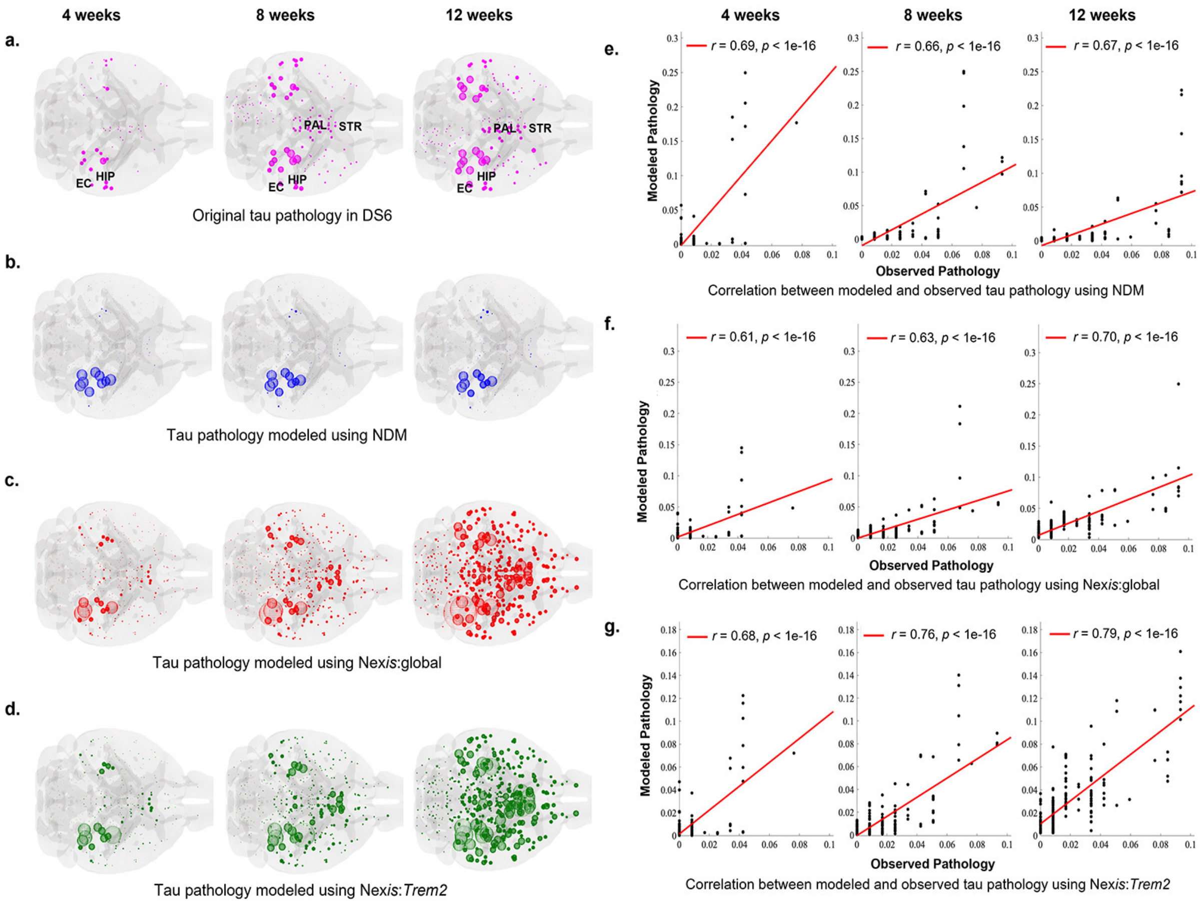

Figure S3. **NDM, Nex*is*:global, and Nex*is*:*Trem2* model fits for dataset DS6.** Panel a depicts empirical tau pathology measured by Kaufman *et al* in dataset DS6 . Panels b, c, and d depict NDM, Nex*is*:global, and Nex*is*:*Trem2* modeled tau pathology progression, respectively, in DS6 at 4, 8, and 12 weeks post hippocampal injection. Panels e, f, and g are scatter-plots depicting the correlation between empirical and ND-modeled (e), Nex*is*:global-modeled (f), as well as Nex*is*:*Trem2*-modeled (g) tau pathology, respectively, at 4, 8, and 12 weeks post hippocampal tau seeding. (HIP: hippocampal area, EC: entorhinal cortex, PAL: pallidum, STR: striatum.)

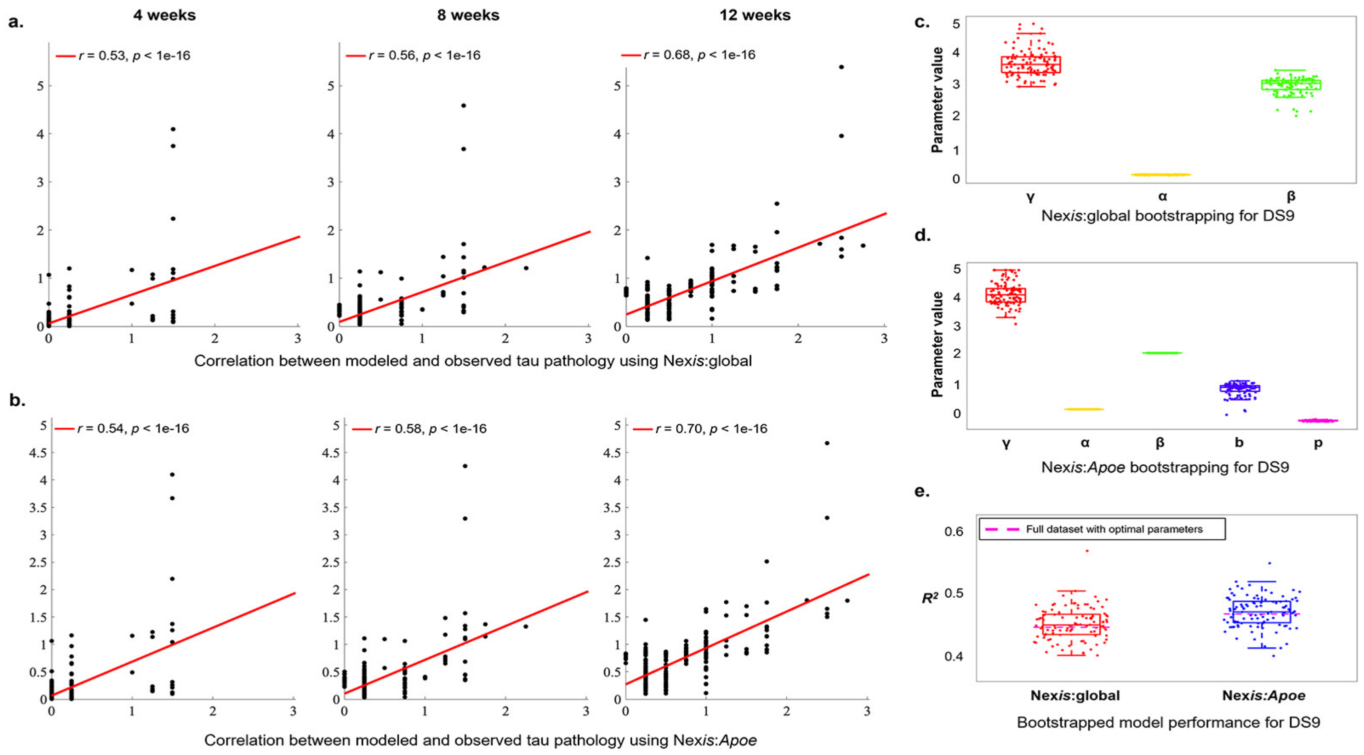

Figure S4. **Nex*is*:global and Nex*is*:*Apoe* model fits for dataset DS9.** Panels a and b depict the Nex*is*:global and Nex*is*:*Apoe* modeled tau pathology progression, respectively, in DS9 dataset at 4, 8, and 12 weeks post hippocampal seeding. Panels c and d depict the 100-iteration 80% resampled bootstrapped values of both Nex*is* model parameters for DS9. Panel e depicts the comparison between model fits of the 80% resampled bootstrapped dataset with the full dataset. Global parameters: γ (seed rescale), α (intra-regional tau accumulation rate), and β (inter-regional tau spread rate). Microglial parameters: p (microglia-mediated intra-regional tau accumulation rate) and b (microglia-mediated inter-regional tau spread rate). Model fit is depicted by R^2.

10 Tables

Table S1. Nex*is*:microglia models applied to individual microglial homeostasis genes. The Nex*is*:microglia model fits and parameter values for some microglial homeostasis genes are presented for datasets DS4, DS6, and DS9. AIC: Akaike's Information Criteria, BIC: Bayesian Information Criteria, R^2: model fit parameter, α: intra-regional tau accumulation rate; β: inter-regional tau spread rate; p: microglia-mediated intra-regional tau accumulation rate; b: microglia-mediated inter-regional tau spread rate. The parameters are presented as mean with 95% confidence intervals.

		Metric			Parameter value			
Dataset	Model	R^2	AIC	BIC	α	β	p	b
DS4	Nex*is*:*P2ry12*	0.18	231.59	257.79	0.32 [0.32 - 0.32]	1.46 [1.32 - 2.46]	-0.13 [-0.16 - -0.11]	0.47 [-0.66 - 0.87]
	Nex*is*:*Cx3cr1*	0.19	222.69	248.89	0.26 [0.17 - 0.32]	1.32 [1.32 - 1.32]	0.02 [-0.24 - 0.36]	1.71 [0.57 - 2.87]
	Nex*is*:*Fcrls*	0.19	229.19	255.39	0.19 [0.17 - 0.32]	1.95 [1.32 - 2.46]	0.51 [-0.59 - 0.77]	0.88 [-1.19 - 4.17]
	Nex*is*:*Olfml3*	0.19	227.12	253.32	0.31 [0.24 - 0.32]	1.36 [1.32 - 2.46]	-0.15 [-0.20 - 0.02]	0.65 [-0.68 - 1.10]
	Nex*is*:*Hexb*	0.18	233.17	259.37	0.19 [0.17 - 0.32]	2.22 [1.32 - 2.46]	0.11 [-0.11 - 0.16]	-0.13 [-0.46 - 0.61]
	Nex*is*:*Siglech*	0.19	225.02	251.22	0.17 [0.17 - 0.18]	2.43 [2.05 - 2.46]	0.17 [0.12 - 0.21]	-0.29 [-0.51 - -0.12]
	Nex*is*:*Sox5*	0.25	183.15	209.35	0.21 [0.17 - 0.24]	2.45 [2.30 - 2.46]	0.12 [0.03 - 0.29]	-1.01 [-1.28 - -0.86]
	Nex*is*:*Jun*	0.18	233.17	259.37	0.32 [0.29 - 0.32]	1.55 [1.32 - 2.11]	-0.11 [-0.14 - -0.06]	0.35 [-0.39 - 0.78]
DS6	Nex*is*:*P2ry12*	0.51	645.14	671.34	0.15 [0.14 - 0.26]	2.41 [2.41 - 2.41]	0.11 [-0.12 - 0.19]	-0.78 [-1.19 - -0.32]
	Nex*is*:*Cx3cr1*	0.47	693.19	719.39	0.18 [0.14 - 0.26]	2.15 [1.29 - 2.41]	0.14 [-0.29 - 0.38]	-0.12 [-2.56 - 1.54]
	Nex*is*:*Fcrls*	0.58	531.15	557.35	0.26 [0.25 - 0.26]	1.31 [1.30 - 1.40]	-0.46 [-0.56 - -0.34]	1.48 [0.26 - 2.37]
	Nex*is*:*Olfml3*	0.50	649.49	675.69	0.14 [0.14 - 0.14]	2.33 [1.29 - 2.41]	0.21 [0.15 - 0.24]	-0.16 [-0.47 - 0.73]
	Nex*is*:*Hexb*	0.49	662.14	688.33	0.14 [0.13 - 0.16]	2.41 [2.41 - 2.41]	0.11 [0.04 - 0.16]	-0.58 [-0.94 - -0.08]
	Nex*is*:*Siglech*	0.47	686.23	712.43	0.25 [0.19 - 0.26]	2.23 [1.29 - 2.41]	-0.13 [-0.21 - 0.04]	-0.66 [-1.05 - -0.24]
	Nex*is*:*Sox5*	0.55	586.34	612.54	0.17 [0.14 - 0.23]	2.03 [1.38 - 2.41]	0.13 [-0.08 - 0.31]	-0.89 [-1.06 - -0.67]
	Nex*is*:*Jun*	0.47	688.38	714.58	0.14 [0.14 - 0.14]	2.30 [1.81 - 2.41]	0.11 [0.01 - 0.17]	-0.53 [-1.31 - 0.08]
DS9	Nex*is*:*P2ry12*	0.48	506.77	532.97	0.15 [0.13 - 0.24]	3.91 [3.91 - 3.91]	0.08 [-0.16 - 0.14]	-0.61 [-0.87 - -0.48]
	Nex*is*:*Cx3cr1*	0.44	547.44	573.64	0.22 [0.13 - 0.24]	3.50 [2.43 - 3.91]	-0.13 [-0.34 - 0.27]	-0.54 [-0.94 - 0.44]
	Nex*is*:*Fcrls*	0.51	476.38	502.58	0.24 [0.21 - 0.24]	2.10 [2.10 - 2.10]	-0.55 [-0.65 - -0.37]	0.20 [-0.27 - 0.50]
	Nex*is*:*Olfml3*	0.45	536.15	562.35	0.13 [0.13 - 0.21]	3.33 [2.10 - 3.91]	0.14 [-0.11 - 0.18]	-0.12 [-0.58 - 0.59]
	Nex*is*:*Hexb*	0.47	520.77	546.96	0.13 [0.13 - 0.18]	3.91 [3.91 - 3.91]	0.10 [-0.03 - 0.12]	-0.43 [-0.63 - -0.31]
	Nex*is*:*Siglech*	0.46	526.74	552.94	0.23 [0.19 - 0.24]	3.91 [3.91 - 3.91]	-0.11 [-0.17 - -0.03]	-0.56 [-0.73 - -0.45]
	Nex*is*:*Sox5*	0.52	457.03	483.23	0.20 [0.13 - 0.23]	3.78 [3.14 - 3.91]	-0.07 [-0.19 - 0.21]	-0.97 [-1.14 - -0.79]
	Nex*is*:*Jun*	0.45	539.44	565.63	0.13 [0.13 - 0.13]	2.99 [2.10 - 3.59]	0.11 [0.06 - 0.13]	0.03 [-0.26 - 0.40]

Table S2. Comparison of model fits for Nex*is*:global, Nex*is*:*Trem2*, Nex*is*:*Apoe*, and Nex*is*:*Trem2:Apoe* for datasets DS4, DS6, and DS9. The Nex*is*:microglia fits using *Trem2* and *Apoe* are compared to Nex*is*:global fits using Fisher's R-to-Z test. AIC: Akaike's Information Criteria, BIC: Bayesian Information Criteria, R^2: model fit parameter, α: intra-regional tau accumulation rate; β: inter-regional tau spread rate; p: microglia-mediated intra-regional tau accumulation rate; b: microglia-mediated inter-regional tau spread rate. The parameters are presented as mean with 95% confidence intervals. *** $p = 0.001$, ** $p < 0.005$.

		Metric			Parameter value			
Dataset	Model	R^2	AIC	BIC	α	β	p	b
DS4	Nex*is*:global	0.17	233.48	250.94	0.25 [0.05 - 0.30]	1.89 [0.05 - 3.84]	-	-
	Nex*is*:*Trem2*	0.20	216.41	242.61	0.17 [0.17 - 0.19]	1.42 [1.32 - 2.46]	0.77 [0.50 - 0.89]	4.25 [0.72 - 5.79]
	Nex*is*:*Apoe*	0.18	232.26	258.46	0.32 [0.27 - 0.32]	1.36 [1.32 - 2.46]	-0.14 [-0.17 - -0.01]	0.75 [-0.62 - 1.32]
	Nex*is*:*Trem2:Apoe*	0.20	216.52	242.72	0.17 [0.17 - 0.19]	1.43 [1.32 - 2.46]	0.76 [0.49 - 0.88]	4.14 [0.69 - 5.69]
DS6	Nex*is*:global	0.46	695.94	713.40	0.20 [0.13 - 0.23]	1.86 [0.23 - 2.61]	-	-
	Nex*is*:*Trem2*	0.60***	523.94	550.14	0.25 [0.21 - 0.26]	1.30 [1.30 - 1.30]	-0.29 [-0.42 - -0.03]	1.33 [0.46 - 2.47]
	Nex*is*:*Apoe*	0.51	647.59	673.79	0.26 [0.26 - 0.26]	1.30 [1.30 - 1.30]	-0.12 [-0.14 - -0.09]	1.57 [0.69 - 1.99]
	Nex*is*:*Trem2:Apoe*	0.60***	524.02	550.21	0.25 [0.21 - 0.26]	1.30 [1.30 - 1.30]	-0.29 [-0.42 - -0.03]	1.32 [0.47 - 2.43]
DS9	Nex*is*:global	0.44	545.09	562.55	0.19 [0.16 - 0.20]	3.01 [2.19 - 3.35]	-	-
	Nex*is*:*Trem2*	0.52**	463.64	489.84	0.22 [0.18 - 0.24]	2.10 [2.10 - 2.10]	-0.31 [-0.45 - -0.10]	0.41 [-0.21 - 0.92]
	Nex*is*:*Apoe*	0.46	527.19	553.38	0.24 [0.24 - 0.24]	2.10 [2.10 - 2.10]	-0.13 [-0.17 - -0.10]	0.90 [0.21 - 1.13]
	Nex*is*:*Trem2:Apoe*	0.52**	463.63	489.83	0.23 [0.18 - 0.24]	2.10 [2.10 - 2.10]	-0.31 [-0.45 - -0.11]	0.40 [-0.22 - 0.91]

Table S3. Nex*is*:microglia models applied to microglial genes involved in phagocytosis and inflammatory response. The Nex*is*:microglia model fits and parameter values for some candidate genes involved in phagocytosis and inflammation are presented for datasets DS4, DS6, and DS9. AIC: Akaike's Information Criteria, BIC: Bayesian Information Criteria, R^2: model fit parameter, α: intra-regional tau accumulation rate; β: inter-regional tau spread rate; p: microglia-mediated intra-regional tau accumulation rate; b: microglia-mediated inter-regional tau spread rate. The parameters are presented as mean with 95% confidence intervals.

		Metric			Parameter value			
Dataset	Model	R^2	AIC	BIC	α	β	p	b
DS4	Nex*is*:*Sorl1*	0.20	221.94	248.14	0.17 [0.17 - 0.17]	2.23 [1.35 - 2.46]	0.16 [0.13 - 0.18]	-0.01 [-0.35 - 0.62]
	Nex*is*:*Clu*	0.19	226.13	252.33	0.17 [0.17 - 0.17]	2.12 [1.21 - 2.25]	0.29 [0.20 - 0.36]	-0.06 [-0.45 - 0.68]
	Nex*is*:*Cd33*	0.18	231.34	257.54	0.17 [0.17 - 0.17]	1.66 [1.25 - 2.14]	0.36 [0.26 - 0.41]	0.60 [-0.32 - 1.75]
	Nex*is*:*Sirpa*	0.19	228.92	255.12	0.24 [0.17 - 0.32]	2.45 [2.44 - 2.46]	0.02 [-0.16 - 0.14]	-0.67 [-0.84 - 0.52]
	Nex*is*:*Aif1*	0.20	218.31	244.51	0.23 [0.18 - 0.27]	2.41 [1.32 - 2.46]	0.23 [-0.11 - 0.59]	-1.20 [-1.50 - 0.59]
DS6	Nex*is*:*Sorl1*	0.58	549.76	575.96	0.14 [0.14 - 0.17]	2.41 [2.41 - 2.41]	0.15 [0.05 - 0.18]	-0.53 [-0.66 - -0.43]
	Nex*is*:*Clu*	0.47	694.93	721.13	0.24 [0.14 - 0.26]	1.50 [1.23 - 2.28]	-0.14 [-0.32 - 0.23]	0.23 [-1.48 - 1.53]
	Nex*is*:*Cd33*	0.48	685.40	711.60	0.25 [0.14 - 0.26]	2.13 [1.57 - 2.28]	-0.18 [-0.33 - 0.35]	-0.07 [-0.95 - 0.51]
	Nex*is*:*Sirpa*	0.58	560.03	586.23	0.14 [0.14 - 0.19]	2.41 [2.41 - 2.41]	0.16 [0.10 - 0.20]	-0.86 [-0.95 - 0.70]
	Nex*is*:*Aif1*	0.47	690.27	716.47	0.24 [0.14 - 0.26]	2.17 [1.30 - 2.41]	-0.22 [-0.59 - 0.57]	-0.54 [-2.21 - 1.03]
DS9	Nex*is*:*Sorl1*	0.51	471.68	497.88	0.15 [0.13 - 0.24]	3.87 [3.62 - 3.91]	0.08 [-0.13 - 0.12]	-0.56 [-0.70 - -0.48]
	Nex*is*:*Clu*	0.44	548.97	575.17	0.21 [0.13 - 0.24]	2.56 [2.01 - 2.99]	-0.09 [-0.23 - 0.16]	0.26 [-0.77 - 1.43]
	Nex*is*:*Cd33*	0.44	548.37	574.56	0.23 [0.19 - 0.24]	3.44 [3.02 - 3.74]	-0.24 [-0.35 - -0.02]	-0.66 [-1.15 - -0.07]
	Nex*is*:*Sirpa*	0.52	456.63	482.83	0.15 [0.13 - 0.22]	3.83 [3.43 - 3.91]	0.06 [-0.11 - 0.12]	-0.86 [-0.92 - -0.79]
	Nex*is*:*Aif1*	0.45	543.25	569.45	0.22 [0.13 - 0.24]	3.59 [2.10 - 3.91]	-0.19 [-0.61 - 0.44]	-0.87 [-1.13 - 0.36]
